# Discovery of novel INK4C small-molecule inhibitors to promote human and murine hematopoietic stem cell *ex vivo* expansion

**DOI:** 10.1038/srep18115

**Published:** 2015-12-18

**Authors:** Xiang-Qun Xie, Peng Yang, Yu Zhang, Peng Zhang, Liping Wang, Yahui Ding, Ming Yang, Qin Tong, Haizi Cheng, Qing Ji, Terence McGuire, Weiping Yuan, Tao Cheng, Yingdai Gao

**Affiliations:** 1 Department of Pharmaceutical Sciences and Computational Chemical Genomics Screening Center, School of Pharmacy; NIH National Center of Excellence for Computational Drug Abuse Research; Drug Discovery Institute; Departments of Computational Biology and Structural Biology, School of Medicine, University of Pittsburgh, Pittsburgh, Pennsylvania 15260, United States; 2 Key Laboratory of Experimental Hematology, Institute of Hematology and Blood Diseases Hospital, Chinese Academy of Medical Sciences and Peking Union Medical College, Tianjin 300020, P. R. China

## Abstract

Hematopoietic stem cells (HSCs) have emerged as promising therapeutic cell sources for high-risk hematological malignancies and immune disorders. However, their clinical use is limited by the inability to expand these cells *ex vivo*. Therefore, there is an urgent need to identify specific targets and effective probes that can expand HSCs. Here we report a novel class of INK4C (p18^INK4C^ or p18) small molecule inhibitors (p18SMIs), which were initially found by *in silico* 3D screening. We identified a lead p18 inhibitor, XIE18-6, confirmed its p18-targeting specificity and bioactivity of promoting HSCs expansion, and then performed structure-activity relationship (SAR) studies by synthesizing a series of analogs of XIE18–6. Among these, compound **40** showed the most potent bioactivity in HSCs expansion (ED_50_ = 5.21 nM). We confirmed that compound **40** promoted expansion of both murine and human HSCs, and also confirmed its p18-targeting specificity. Notably, compound **40** did not show significant cytotoxicity toward 32D cells or HSCs, nor did it augment leukemia cell proliferation. Taken together, our newly discovered p18SMIs represent novel chemical agents for murine and human HSCs *ex vivo* expansion and also can be used as valuable chemical probes for further HSC biology research towards promising utility for therapeutic purposes.

Stem cells are primal cells which were found in most multi-cellular organisms. They are characterized by their ability to self-renew through mitotic cell divisions and to differentiate into a diverse range of specialized cell types. Self-renewal of stem cells is necessary for tissue repair and maintenance of organ integrity in most mammalian systems. Among the many types of stem cells, hematopoietic stem cell (HSC) is one of the most widely studied. HSCs are able to reproduce and differentiate into all kinds of blood cells, including erythroid, myeloid, and lymphoid lineages^1^,^2^,^3^,^4^,^5^. Thus, HSCs have a high therapeutic potential to remedy high-risk hematological malignancies, as well as other diseases of blood-forming cells and the immune system^6^,^7^,^8^.

Although practiced clinically for more than 50 years, the use of HSCs transplantation remains limited by the lack of HSCs sources and inability to expand these cells *ex vivo* for therapeutic needs. Three sources of HSCs for transplantation mainly include umbilical cord blood (UCB), bone marrow (BM) and mobilized peripheral blood (mPB)^5^. Among these, UCB has several clinical advantages, including rapid and convenient availability from numerous CB banks, less stringent criteria for human leukocyte antigen (HLA) matching, lower incidence of severe graft-versus-host disease (GVHD) without compromising graft-versus-leukemia effects, lower risk of viral transmission and the absence of risk to donors^5^. However, the limited dose of hematopoietic stem and progenitor cells (HSCs and HPCs) provided in one CB unit results in a higher incidence of graft failure and delayed recovery of neutrophils and platelets leading to higher risk of bacterial and fungal infections^9^,^10^,^11^.

To overcome this significant restriction against broader use of HSCs, various attempts have been made to expand human UCB HSCs and HPCs *ex vivo* in order to acquire a larger number of transplantable HSCs/HPCs. Among these attempts, small molecules targeting specific signaling pathways and mechanisms are becoming increasingly accessible. We have also demonstrated that small chemical molecules have distinct advantages in manipulating stem cell fates and can be used as valuable chemical probes for HSC biology studies^12^. These types of approaches have played essential roles in stem cell research and regenerative medicine^13^,^14^; however, these efforts have not resulted in sufficient HSCs expansion in clinical trials. In addition to this limitation, transplanted HSCs may also directly or indirectly contribute to the development of leukemia^15^,^16^.

Among various cell signaling proteins, the INK4 family protein, INK4C or p18^INK4C^ (hereafter referred to as p18), is a critical regulator of the early G1-phase of the cell cycle through the inhibition of CDK4/6^17^. Research by us and others has established p18 as a key player in HSCs self-renewal^18^,^19^ and also an important inhibitor of stem/progenitor cell self-renewal in other tissue types, including the lungs and the brain^20^,^21^. Specifically, we demonstrated a significant increase of HSCs self-renewal in the absence of p18^19^. Furthermore, we showed that the absence of p18 was able to overcome the exhaustion of HSCs in serial transplantation over the course of three years^18^. Importantly, HSCs are not the direct targets of spontaneous leukemic transformation in p18-null reconstituted mice, and overgrowth of p18-null HSCs did not lead to a leukemic phenotype^22^. Moreover, our recent study suggests that leukemic transformation is inhibited by over-expression of p18 in murine embryonic stem cells, but not in adult stem cells or tumor cells^23^. Our most recent results revealed that p18 is a more potent inhibitor of HSCs self-renewal than p27 in mouse models^12^. We further identified that p18 chemical inhibitors could specifically block the bioactivity of p18 protein, and demonstrated that the lead compounds were able to expand functional murine HSCs *ex vivo*^12^. Taken together, these studies suggested that p18 was an attractive drug target for specific small molecule chemical inhibitors to manipulate HSCs with potential for therapeutic purposes.

In the present study, we report a new class of small molecule inhibitors targeting p18 (p18SMIs), which were identified, optimized and validated through a systematic and interdisciplinary pipeline. The representative compounds display strong and improved bioactivity in stimulating HSCs (ED_50_ < 10 nM). These agents do not exhibit any sign of inducing toxicity in a murine bone marrow-derived 32D cell line. More importantly, the best of these derivatives, compound **40** (ED_50_ = 5.21 nM), shows no activity in promoting the proliferation of leukemia cells. Our mechanistic studies reveal that compound **40** could selectively promote HSCs division by inhibiting p18, thereby activating CDK4/6. Overall, our study leads to the identification of three new potent derivatives (ED_50_ < 10 nM) and also confirms that compound **40** is a novel and effective p18 inhibitor in promoting HSCs expansion in both murine and human models.

## Results

### Virtual Screening for p18 Small Molecule Inhibitors (p18SMIs)

**Figure 1:**
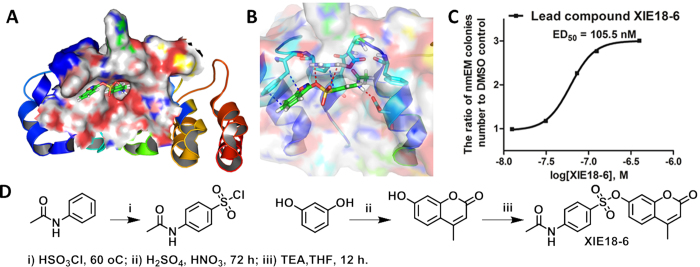
Identification of the lead compound XIE18-6 as p18 small molecule inhibitor (or p18SMI). (**A**) Docked pose of XIE18-6 in the p18 binding pocket: protein surface is colored by lipophilic potential; compound XIE18-6 is shown in different atom types. (**B**) Proposed interactions between XIE18-6 and p18 residues: p18 is shown as cartoon; residues of p18 are shown as different atom types; XIE18-6 is shown in green sticks and different atom types; hydrogen bonds and π-π interaction are represented as red and blue dashed lines, respectively. (**C**) Bioactivity exploration of XIE18-6 over HSCs using the single cell culture assay. Data is mean ± SD for all experiments of two or more and were performed in duplicate or triplicate. (**D**) Synthesis of XIE18-6. Reagents and conditions are as follows: i) HSO_3_Cl, 60 °C; ii) H_2_SO_4_, HNO_3_, 72 h; iii) TEA, THF, 12 h.

Increasing evidence indicates that p18SMIs are an effective technique for manipulating HSCs expansion. Indeed, we recently reported a p18SMI which could expand HSCs with an ED_50_ of 3.61 μM^12^. We sought to extend upon this seminal finding and develop a more potent agent to overcome the intrinsic limitation and low throughput of current HSCs bioassays. Towards this end, we first identified putative p18SMIs by modeling the 3D structure of the p18 protein and determined its binding surface cavity based on the X-ray co-crystal structure of the p18/CDK6 complex (PDB entry: 1G3N), using our reported protocols (Fig. 1A)^24^,^25^,^26^. Both the predicted 3D structure of p18 and its binding model were then used to identify p18SMIs by virtual screening and molecular docking studies. A chemical library of NCI2010 compound database was *in silico* screened using the Surflex-Dock program in Sybyl-X 1.3. The 200 top-ranked hit molecules with docking scores greater than 7.5 were subjected to manual docking inspection according to three criteria: (1) at least three hydrogen bonds between ligand and p18 should be formed; (2) a conserved hydrogen bond with Arg39 or Asp76 of p18 should exist; and (3) diversity of scaffolds, as well as drug-like properties, should be considered. Based on these criteria we defined a subset of 22 compounds which were commercially obtained from NCI, and tested for their ability to promote HSCs expansion.

During the 3D docking studies, using the Surflex-Dock GeomX module, different binding poses or conformations of each ligand, were generated. A representative binding pose of protein/inhibitor interactions is illustrated in Fig. 1A, in which the lead compound, XIE18–6, bound to a defined p18 pocket surrounded by residues R39, V44, V45, G48, D67, D76, R79 and D84. These p18 residues were known to form strong H-bonds with CDK6^12^. Additional residues identified in the binding cavity are F37, L47, T69, F71, A72 and V73. Overall, the docking poses suggested favorable interactions between XIE18–6 and p18 residues. We observed several extensive hydrogen bonds involving the amide NH group of XIE18–6 with the p18 residue D76 (1.99 Å), the sulphonyl O=S=O with residue R39 (2.74 Å), and the sulfonate O-SO_2_ with residue R39 (2.85 Å), as well as a π-π interaction between XIE18–6 lactone ring and p18 residue F37 (Fig. 1B). The computed binding mode suggested that XIE18–6 had strong interactions with p18 and should be expected to disrupt the interaction of p18 with CDK6.

### Lead Compound XIE18–6 Promotes HSC Expansion

To confirm the screening hits and provide sufficient amount of high-purity samples for biological exploitation, XIE18–6 was chemically synthesized, purified and characterized for quality control checking. As illustrated in Fig. 1D, *N*-phenylacetamide was reacted with chlorosulfonic acid to give the first intermediate, 4-acetamidobenzenesulfonyl chloride. Starting from a highly activated phenol (resorcinol) and a carboxylic acid (ethyl acetoacetate) containing a β-carbonyl group, another coumarin intermediate was synthesized using a Pechmann condensation reaction, which was performed under mild condition using H_2_SO_4_ as the acid catalyst^27^. The coupling reaction between two intermediates was conducted in tetrahydrofuran (THF) using triethylamine (TEA) as the base. This reaction yielded the final compound XIE18-6, which was further purified by flash column chromatography^28^ and characterized by NMR and MS.

We biologically assessed the 22 putative p18SMI compounds for their ability to maintain HSCs stem cell characteristics, to be specific self-renewal and multi-lineage differentiation, using single cell cultures^29^. It has been reported that cells giving rise to neutrophils (n), macrophages (m), erythroblasts (E) and megakaryocytes (M) (or nmEM) cells are a major subset with superior capacity of proliferation among HSCs^29^. Thus, we employed single HSC culture to assess p18SMI compounds’ effect upon HSCs. CD34^−^LKS cells from C57BL/6 mice were individually deposited into a 96-well plate (one cell/well). The single cell was cultured for 14 days in 100 μL of serum-free medium (Gibco) containing 100 ng/mL of mouse stem cell factor (SCF), 10 ng/mL of mouse interleukin-3 (IL-3), 25 ng/mL of human thrombopoietin (TPO), 2 U/mL of human erythropoietin (EPO) and differing concentrations of the p18SMI compound. Cells were cultured 14 days for *in vitro* colony formation and colonies were then cytospined and analyzed by flow cytometry to test the subpopulation of cultured cells. Neutrophils (n), macrophages (m), erythroblasts (E) or megakaryocytes (M) were identified by the cell marker of Gr-1, Mac-1, Ter-119 and CD41 using flow cytometry. The wells from each group that generated all lineages (nmEM) were counted and the ratio to the control group (DMSO) was calculated. The results suggested that most compound treatment could significantly increase the number of wells that could be detected with nmEM colonies. And among all the tested compounds, XIE18–6 had a prominent effect on promoting HSC expansion with an ED_50_ of 105.5 nM (Fig. 1C, Fig. S4).

### Lead Optimization and Modification via Structure-activity Relationship (SAR) Studies

To broaden the SAR and to improve the potency of XIE18–6, we conducted structural modification studies to design and synthesize seven series of analogues of XIE18–6, which are represented by 41 analogues that are reported herein (the chemical structures and bioactivity are summarized in Supplemental Tables 1–7). The synthetic routes used to obtain the target XIE18-6 analogues are illustrated in Fig. 1D and Fig. S3. As described above, the single-cell *in vitro* culture assay was used to determine the potential of the analogues to promote HSCs expansion^29^. Among the illustrated 41 novel analogues, 14 promoted the expansion of HSCs with a low nanomolar ED_50_ and improved water solubility for cell permeability.

**Figure 2:**
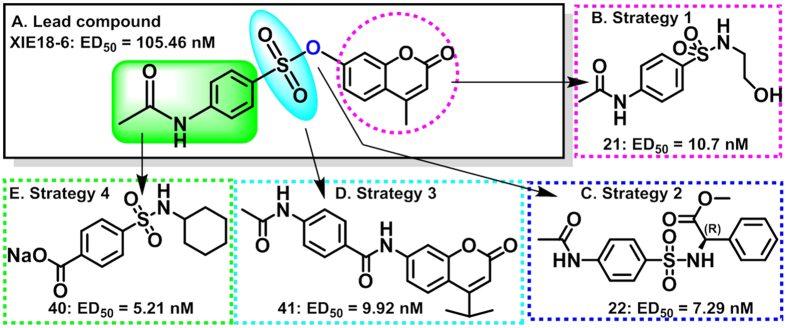
Medicinal chemistry optimization and modification through SAR studies of lead compound XIE18-6. (**A**) Lead compound XIE18-6; (**B–E**) Strategies of lead modification and representative analogues.

Starting with the fused ring moiety, our strategy to modify XIE18–6 for lead optimization focused on its lactone ring (Fig. 2). We removed the methyl group or increased the size of the -CH_3_ group on the lactone ring by substituting it with an isopropyl group. This yielded compounds **1** and **2**, which showed similar activity in HSCs, compared to XIE18–6 (Table S1: XIE18–6, ED_50_ = 105.5 nM; **1**, ED_50_ = 97 nM; and **2**, ED_50_ = 61 nM). Replacement of XIE18–6’s lactone ring with different aromatic rings bearing diverse substituents produced 12 additional compounds (Table S2).When compared to compound XIE18–6 (ED_50_ = 105.5 nM), replacement of the lactone ring with smaller phenethylamino or benzylamino groups (compounds **3**–**8**) dramatically decreased the compound’s activity (ED_50_ > 1 μM) in HSCs. The results of compounds **3**–**8** indicated that as the substituted lactone ring became smaller, the activity decreased. Based on this observation, we further chemically decreased the size of the substituent and reduced the distance between two aromatic rings in the molecule. We replaced the lactone ring with a substituted anilino group and synthesized compounds **9**–**14**. Among them, compound **12** exhibited a 4-fold higher activity in HSCs with an ED_50_ of 25.9 nM. This indicated that the lactone ring may not be essential for activity. In addition to replacing the lactone ring with an aromatic ring, we replaced it with different saturated groups and synthesized another set of seven compounds (**15**–**21**, Table S3). This modification generated compound **21**, which had improved activity (ED_50_ = 10.7 nM and 10-fold superior toXIE18–6). Meanwhile, we replaced the lactone ring with two amino acids bearing a different R-configuration or S-configuration (Table S4). Compound **22** (R-configuration) exhibited improved activity with an ED_50_ of 7.29 nM, while compound **23** (S-configuration) showed a complete loss of activity in promoting HSCs expansion (ED_50_ > 20 μM). The activity of compound **22** is over 1,000-fold higher than that of compound **23**. This result indicated that the ability of a compound to promote HSCs expansion was very sensitive to the amino acid configuration. This was an interesting result and was the basis for further modification and SAR studies.

We next evaluated the effect of structural modifications on the benzene ring. To do so, we first retained the lactone ring or replaced it with a saturated ring to synthesize two series of compounds (Table S5: compounds **24**–**31** and Table S6: compounds **32**–**39**). Retaining the lactone ring, we replaced the acetamidophenyl group with a linear chain or a benzyl group, producing two compounds. Although they both showed decreased activity in HSCs, the decrease was greater in compound **24** treatment (**24**, ED_50_ = 4.14 μM and **25**, ED_50_ = 0.92 μM). Meanwhile, we introduced different groups to the benzene ring and synthesized six other compounds (**26**–**31**). Replacing the acetamide group with fluorine, *iso*-propyl, or carboxyl decreased the activity (compound **26,** ED_50_ = 0.79 μM; compound **30,** ED_50_ = 1.93 μM; and compound **31,** ED_50_ = 6.72 μM, respectively). Replacing the acetamide group with methyl, methoxyl or chlorine increased the activity (compound **27,** ED_50_ = 0.021 μM; compound **28,** ED_50_ = 0.060 μM; and compound **29,** ED_50_ = 0.078 μM, respectively). Compound **27** featured the methyl group and showed a 5-fold improvement in activity compared to XIE18–6. These results indicated that the acetamide group on the benzene ring may not be essential for HSCs expansion. However, an aromatic benzene ring had an important role in maintaining better expansion of HSCs. We then synthesized eight additional compounds by replacing the lactone ring with a saturated ring (Table S6: compounds **32**–**39**). Among these compounds, three showed nanomolar ED_50_ activity, including the best in this series, compound **37** (ED_50_ = 10 nM), which bore an *iso*-propyl group. These results indicated that the lactone ring might not be essential for activity and could be replaced by another hydrophobic ring. Interestingly, compound **39**, with a carboxyl group, showed similar activity to XIE18–6. We then converted this carboxyl group to sodium (Na^+^) salt, which increased the solubility of a compound. This strategy produced the best XIE18–6 analogue, compound **40**, which showed a 20-fold higher activity in HSCs with an ED_50_ of 5.2 nM (Table S6).

To explore whether the sulphonyl group of XIE18–6 was essential for HSC expansion activity, we replaced the sulphonyl group with a carboxyl group and created compound **41** (ED_50_ = 9.92 nM, Table S7). This modification resulted in significantly increased expansion activity, which suggested that the sulphonyl group was not essential and that substitution with a carboxyl group might be a good strategy for further SAR study.

Overall, the medicinal chemistry SAR studies led to 14 top compounds with an ED_50_ of 100 nM or lower. Among these, compound **40** (sodium salt) showed the highest bioactivity and improved drug-like solubility properties. On the basis of activity and drug-likeness attributes, compound **40** was selected as the best candidate for further biological study, described below.

### The Number of Phenotypic Long Term (LT)-HSCs Increases after Treatment with p18SMIs

**Figure 3:**
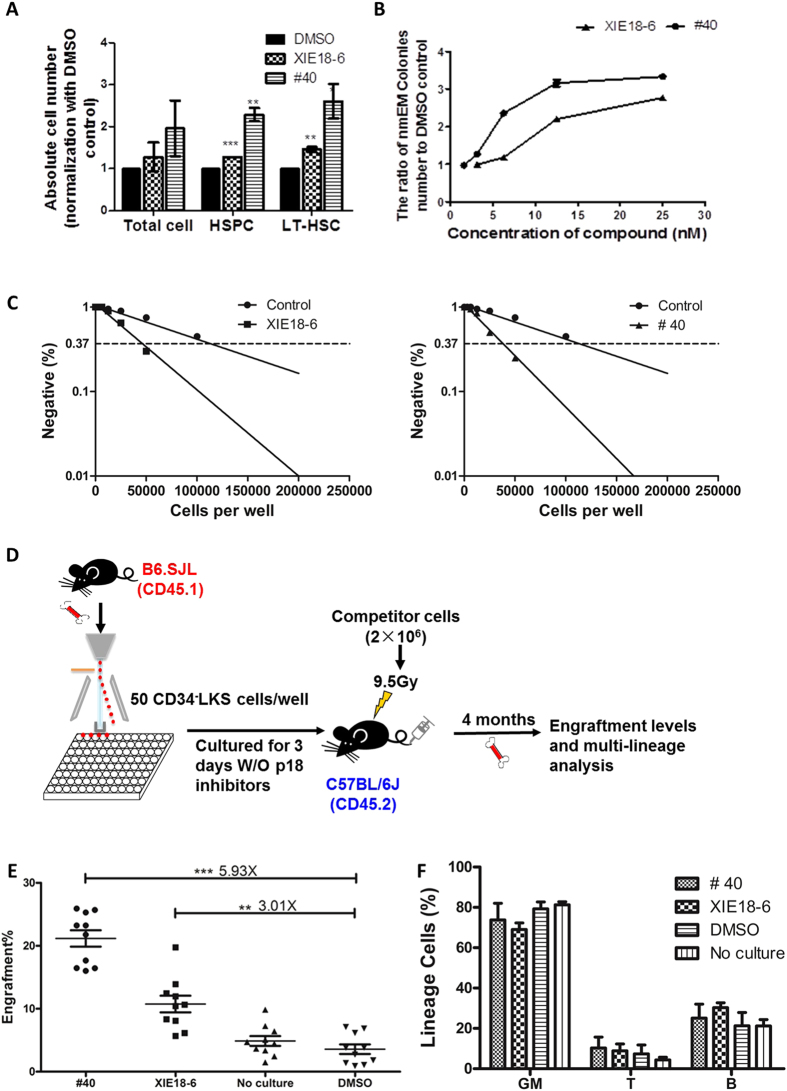
Treatment of c-Kit-enriched murine BM cells with p18SMI compounds enhances HSC expansion. (**A**) The average number of total cells, HSPCs (Lin^−^Sca1^+^) and long term (LT)-HSCs (Lin^-^Sca1^+^CD48^−^CD150^+^) were analyzed by flow cytometry. (**B**) Single CD34^−^LKS cell were deposited into each well of a 96-well plate, along with differentiation medium plus different concentrations of p18SMI compounds to show the dose-response effect of p18SMI compounds. (**C**) CAFC-LDA assay of cells treated with XIE18-6, compound **40** and DMSO to reveal the frequency of primitive HSCs. Above data (**A**–**C**) is mean ± SEM for all experiments of two or more and were performed in duplicate or triplicate. (**D**) Experimental design for transplantation (n = 10). CD34^-^LKS cells were sorted into 96-well plates at 50 cells/well and cultured for 3 days with cytokine plus 20 μM of compounds or DMSO. Cells from each well were mixed with 2 × 10^6^ freshly isolated bone marrow MNCs and co-transplanted by tail-vein injection into lethally irradiated CD45.2 recipient mice. 16 weeks after transplantation, bone marrow MNCs were stained to determine the engraftment level of donor cells. Bone marrow MNCs were also stained for lineage analysis. Multi-lineage differentiation was examined by flow cytometry. GM, T and B indicate lineages for myeloid, T and B cells, respectively. (**E**,**F**) The engraftment levels at 16 weeks after primary transplantation, and multi-lineage differentiation profile. Data is mean ± SEM for all experiments (***p < 0.001, **p < 0.01).

To investigate the effect of p18SMI compounds on murine HSCs, c-Kit-enriched mouse BM cells were cultured for 5 days with media containing cytokines and the p18SMI compounds. Despite a slight increase of total cellularity (total cell) for the group treated with compound **40** (Fig. 3A, Fig. S2), the absolute number of Lin^-^Sca-1^+^ hematopoietic stem/progenitor cells (HSPC) increased 2.29-fold. Lin^−^Sca-1^+^(LS) cells were further analyzed using a combination of CD150 and CD48 (SLAM code) biomarkers for HSC identification. CD150^+^ CD48^−^KSLs (hereafter referred to as SLAM^+^) cells are enriched for long term (LT)-HSCs. Interestingly, the number of LT-HSCs increased 2.61-fold after treatment with compound **40**. Representative flow plot figures were shown in Fig. S3. These data suggested that compound **40** could selectively act on HSCs and increases the number of phenotypic LT-HSCs in bulk culture.

Next, we assessed the ability of compound **40** to increase the number of HSCs and maintain the stem cells characteristics using the single cell culture (nmEM colony assay), as described above^29^. After a 14-day treatment with compound **40**, the number of nmEM colonies in p18SMI compound-treated group increased in a dose-dependent manner (Fig. 3B, Fig. S4). Exposure to compound **40** at 12.5 nM led to a 3.16-fold increase of nmEM colonies as compared to the control group. These data clearly indicated that compound **40** induced proliferation, but not differentiation of HSCs.

### Functional LT-HSC Populations Increase upon Exposure to p18SMI in Animal Engraftment

We also investigated whether the increased number of phenotypic SLAM^+^LT-HSCs in short-term culture assays was correlated with an increase in functional LT-HSCs. Cobblestone area forming cells (CAFC) assay combined with limiting-dilution assays (LDA) were performed to measure the frequency of HSCs^19^,^22^,^30^. Compared with the control group (HSC frequency is 1 in 112,559 of the original plated cells), the frequency of HSC at day 35 (indicating the activity of hematopoietic reconstitution capacity) was 2.7-fold and 3.0-fold higher in the cultures treated with XIE18–6 (HSC frequency is 1 in 42,490 of the original plated cells) and compound **40** (HSC frequency is 1 in 37,524 of the original plated cells) (*p* < 0.001, Fig. 3C), respectively. These results confirmed that XIE18–6 and compound **40** significantly increased the frequency of HSC in culture, suggesting these compounds positively affected HSCs.

The increased frequency and total number of functional LT-HSCs after treatment with p18SMI compounds was further confirmed by competitive bone marrow transplantation (cBMT) assay (Fig. 3D). CD34^-^LKS cells were first sorted and cultured w/o p18SMI compound for 3 days. Then the cultured cells were gathered and co-transplanted into lethally irradiated mice with competitor cells. 16 weeks after transplantation, flow analysis of bone marrow of recipient animals revealed a 3.01-fold increase in the engraftment of the group treated with XIE18–6 and a 5.93-fold increase in the group treated with compound **40** compared with the DMSO control group (*p* < 0.01, n = 10, Fig. 3E, Fig. S5). Similar increase could also be detected in p18SMI compound treated group when compared with the uncultured group (4.34-fold increase in compound 40 group and 2.20-fold increase in XIE18–6 group, *p* < 0.01, n = 10). Moreover, the engraftment of the group treated with compound **40** was double of that of the group treated with XIE18–6. These results further confirmed that XIE18–6 and compound **40** significantly stimulated the growth of LT-repopulating HSCs *ex vivo*. Importantly, treatment with either XIE18–6 or compound **40** did not cause predominant growth of any specific lineages compared with the control group (Fig. 3F, Fig. S5).

### p18SMIs Promote Human HSC Expansion *ex vivo*

**Figure 4:**
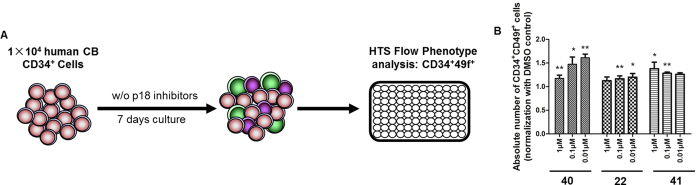
Confirmation of p18SMIs promoting expansion of human hematopoietic stem cells *ex vivo*. (**A**) Experimental design. (**B**) Dose response assay assessing the effective concentrations of various p18SMIs (from primary screening hits) for their ability to promote proliferation of human HSCs. Data is mean ± SEM for all experiments of two or more and were performed in duplicate or triplicate. (***p < 0.001, **p < 0.01, *p < *0.05*).

Based on our study of murine HSCs, we further tested whether these p18SMIs could expand human cord blood CD34^+^ cells in *ex vivo* culture. Here we chose CD34 (the most commonly used marker for human HSCs) and CD49f (revealed as a specific marker for human HSCs^31^) as the primary screening markers of human HSCs. We cultured human cord blood CD34^+^ cells for 7 days in HSCs expansion media in the presence of 200 nM of p18SMIs, and then analyzed the percentage and absolute number of CD34^+^CD49f^+^ cells by flow cytometry. We screened 40 p18SMIs, of which14 compounds could increase the number of CD34^+^CD49f^+^ cells compared with the control group (Fig. S6). Compound **40** was confirmed as our best hit (>30% increase in the absolute number of CD34^+^CD49f^+^ cells) and showed potent bioactivity in expanding human HSC *ex vivo*. Then we conducted a concentration gradient assay on top three p18SMIs with ED_50_ less than 10 nM (compounds **22**, **40** and **41**). The results suggested that the expansion of human HSCs by these p18SMIs is concentration related. Only in specific doses could they show their expansion effect on human HSCs (Fig. 4B).

### p18SMIs Stimulate HSCs Division by Inhibiting p18 and thereby Activating CDK4/6

**Figure 5:**
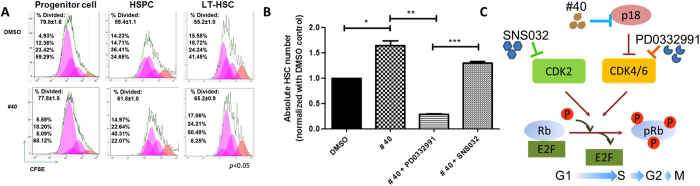
p18SMI compounds increase HSC proliferation through the p18-CDK4/6 pathway in LT-HSC. (**A**) CFSE analysis of bone marrow cells cultured with cytokine plus p18SMI compound to assess the number of cell divisions. Top percentage, first generation; second percentage, second generation; third percentage, third generation; and bottom percentage, fourth generation. **(B)** Specific dependence of compound **40** on CDK4/6, rather than CDK2. (**C**) A model of cell-cycle regulation by p18 and CDKs. p18SMI-induced HSC expansion acts through p18-CDK4/6 pathway in LT-HSC. Data is mean ± SEM for all experiments of two or more and were performed in duplicate or triplicate.

We have previously shown that deficiency of p18 may enhance HSCs expansion by inducing primitive phenotypes after each cell division *in vivo*^19^. To obtain direct evidence that compound **40** promotes primitive phenotypes after each cell division, we measured cell divisions in distinct immunophenotypically-defined populations of cells treated with p18SMI compound. 5- (and 6-) carboxy-fluorescein diacetatesuccinimidylester (CFSE) dye was used to label the c-Kit-enriched BM cells before culture. Surface markers for progenitor cells, HSPCs and LT-HSCs were used to co-stain the BM cells that were harvested 5 days after treatment with the p18SMI compound. The number of initial cell divisions was measured based on the intensity of CFSE in each cell population. Consistent with previous phenotype results, little effect on the progenitor cells and HSPCs populations was observed (Fig. 5A). While there was a significant increase in the number of divided cells that retained the same phenotype in the LT-HSCs population (65.2%) compared to the control group (55.2%). This 10% increase led to a notable difference due to that stem cells divided less than four times in 72 hours, as seen in Fig. 5A. In good agreement with the phenotypic analysis of the hematopoietic cells (Fig. 3A), these data also suggested a selective effect of compound **40** on the primitive cells. As shown in Fig. 5A, the majority of CFSE was retained in the fourth versus third cell generation for populations cultured with DMSO and compound **40**, respectively. This indicated that the enlarged HSC pool in the compound **40**-treated group was a result of more cell division in primitive LT-HSCs but not accelerated exhaustion.

To assess a direct role for p18-CDK6 signaling in HSCs expansion induced by compound **40**, we used the CDK4/6-specific inhibitor PD0332991 to block CDK6 activation. As a control, we blocked CDK2 with SNS032, a CDK2 selective inhibitor. Previously, we have reported that both PD0332991 and SNS032 could not maintain HSCs population due to their CDK inhibition effect and further induction of cell cycle arrest^12^. So here we wanted to explore whether p18SMI compound’s HSCs expansion effect was abrogated by CDK4/6 inhibitor or CDK2 inhibitor. Compound **40** failed to increase the number of LT-HSCs in the PD0332991-treated group, but it still enhanced the number of LT-HSCs in the SNS032-treated group, suggesting that compound **40** was not mediated through CDK2 but rather through the CDK4/6 pathway (Fig. 5B,C).

Collectively, these data provided strong evidence that compound **40** promoted HSCs to enter into the cell cycle by specifically inhibiting p18 and thereby activating CDK4/6, but not CDK2.

### Cytotoxicity and Tumorigenesis Assessment of Top p18SMIs

**Figure 6:**
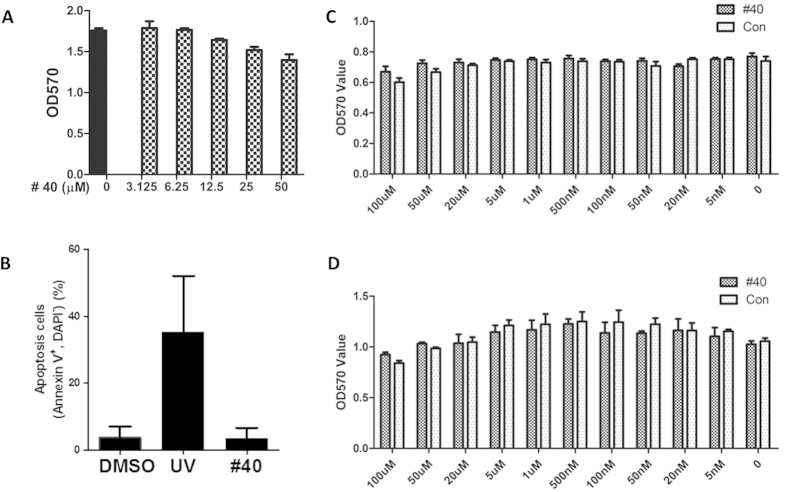
Cytotoxicity assessment of top p18SMI compounds. (**A**) Cell proliferation assay was performed on 32D cells to evaluate general cytotoxicity. The cell survival was determined by the MTT assay. (**B**) Apoptosis statistics analysis of cultured c-Kit enriched BM cells which were cultured with cytokine plus 20 μM compound **40** or DMSO. Uncultured bone marrow cells were irradiated by UV to serve as positive control. Apoptosis was checked by Annexin V staining on hematopoietic cells. (**C**,**D**) Proliferation effect of compound **40** on leukemia cell lines KG-1 and KG-1a. The cell survival was determined with the MTT assay. Data is mean ± SEM for all experiments of two or more and were performed in duplicate or triplicate.

Having confirmed that our newly discovered p18SMI compounds showed promising activity in promoting HSCs expansion, we then investigated their cytotoxicity profile, using different cell types in order to examine whether the top compounds have a favorable therapeutic index. First, a cell-proliferation assay was performed on the immortalized myeloblast-like cell line 32D cells to evaluate general cytotoxicity. After treatment of 32D cells with p18SMI compounds, compound **40** did not show any cytotoxic effects at a high concentration of 25 μM. No significant difference was found and only slight decreases in cell viability were observed at a high concentration of 50 μM, which was thought to be due to cytotoxicity of the solvent DMSO (Fig. 6A). We also evaluated cytotoxicity using primary hematopoietic cells. We observed that, compared with the negative control group, compound **40** did not increase apoptotic cell death at the effective concentration (Fig. 6B, Fig. S7).

With confirmation of compound **40** promoting the expansion of HSCs, we further examined whether it might also increase the risk of malignant diseases. In order to determine the effect of compound **40** on hematological tumorigenesis, proliferation of both leukemia cell lines (KG-1 and KG-1a) and myeloma cell lines (MM.1S and RPMI 8226) was further investigated by a standard MTT assay. Importantly, compound **40** did not enhance KG-1 and KG-1a cells at any concentration (Figs 6C,D, S1), nor myeloma cell lines at 5 μM. These results indicated favorable therapeutic indexes of our compound and without promoting proliferation of leukemia cells as well as myeloma cells.

## Discussion

It is known that stem cells are capable of self-renewal and multi-lineage differentiation, thereby offering a major cellular source for regenerative and transplant medicine. A classic example is HSCs transplantation for the treatment of immune deficiencies, congenital disorders and malignant diseases^32^. Despite the success of HSCs transplant medicine, the full therapeutic potential of HSCs has not yet been developed. There is an urgent need to discover a novel target and an effective probe for HSCs expansion. Recently, several small molecules, such as StemRegenin1 (SR1) and UM171, have been reported to increase HSCs *ex vivo* expansion. However, the underlying mechanisms of these compounds are still unclear, which greatly limits the application of these compounds in clinical treatment and might lead to unexpected side effects. The ultimate success of HSC expansion *ex vivo* and efficient repopulation of the hematopoietic/immune system *in vivo* will require potent and target-specific biological or chemical agents.

p18, a member of the INK4 CDK inhibitor (CKI) family, regulates the G1-phase of the cell cycle by inhibiting CDK4/6^33^. We previously demonstrated a significant increase of adult HSCs self-renewal in the absence of p18^19^. Subsequently, we showed that p18 absence overcame the exhaustion of HSCs in serial transplantation over the course of three years^22^. The role of p18 in HSCs appears to be distinct, as p21 and p18 have opposite effects^22^ and p16 has an age-dependent effect on the durability of HSCs *in vivo*^34^. A unique and relatively specific role of p18 in HSCs was also indicated by studies from other groups. Evidence from the laboratory of Connie Eaves *et al.* (2007) indicated that p18, but not other CKIs, mediates the higher responsiveness of fetal HSCs to SCF, thereby explaining their higher potential for self-renewal than HSCs from adult bone marrow^35^. Margaret Goodell’s team demonstrated that p18, but not p19, p21, nor p27, mediates the effect of Nurr1, a nuclear receptor transcription factor, in maintaining HSC quiescence^36^. All these data make p18 an attractive target for developing specific small-molecule inhibitors in order to manipulate HSC self-renewal.

Based on the protein structure and binding surface cavity of p18, we conducted a 3D database docking virtual screening to obtain virtual p18 inhibitors. Top compounds available from NCI or commercial sources were further confirmed for their ability to promote the expansion of HSCs. Our discovered lead compound, XIE18–6, exhibits biological effect on HSCs expansion after a short exposure in culture. XIE18–6 synthesis involves only a few steps and is therefore amenable to further application as a pharmaceutical lead. To broaden the SAR and to increase the potency of XIE18–6, seven series of p18SMI compounds were designed, synthesized and tested for their ability to induce HSCs growth. Among these, representative compounds, such as **22**, **40**, and **41**, showed desirable activity in HSCs with ED_50_ < 10 nM. Furthermore, the best compound, compound **40**, was not cytotoxic to normal 32D cells or HSCs. More importantly, compound **40** had no effect on leukemia cell lines and myeloma cell lines, even at a high concentration (20 μM).

Our present study demonstrates that compound **40** enhanced the expansion of HSCs. Compound **40** enlarged the pool size and the number of cycling cells in LT-HSCs, but not HSPCs nor progenitor cells. Consistent with our studies on murine HSCs, our preliminary tests on human HSCs confirmed that compound **40** could promote HSCs expansion during *ex vivo* culture. We found that the engraftment advantage of compound **40** was not due to predominant outgrowth of a single lineage or less restricted HSPC pools, but rather due to enhancement of expansion in primary LT-HSCs. Therefore, our data suggested a relatively specific effect of p18 inhibition on HSCs. A plausible mechanism for HSCs expansion may be the increase of the division rate of LT-HSCs. Furthermore, the direct role of compound **40** in HSC expansion strongly indicated that compound **40** promoted HSCs division by inhibiting p18 and activating CDK4/6, not CDK2. Taken together, our study raises the exciting possibility that compound **40** can be developed further and used as a therapeutic drug to promote the expansion of HSCs *ex vivo*.

## Methods

The study has been approved by University of Pittsburgh and Institute of Hematology. All methods used in this study were carried out in accordance with the approved guidelines and all experimental protocols were approved by University of Pittsburgh and Institute of Hematology.

### Molecular modeling

3D structures of the p18 protein were modeled based on the X-ray crystal structures of free p18 (PDB entry: 1IHB) and the p18/CDK6 complex (PDB entry: 1G3N)^37^,^38^ using our reported protein modeling protocol^25^,^26^. The putative p18 binding cavities were defined using a solvent-accessible surface calculation algorithm^24^ based on the interface contacting residues known from X-ray crystallography and site-directed mutations. 3D chemical database virtual screening was carried out using our reported docking virtual screening/CScore method^24^ to identify small molecules that can interfere with p18/CDK6 binding. All computations were performed on a dual-core dual-CPU Xeon-based HPCC 30-processor Dell cluster, loaded with the Tripos Sybyl molecular modeling package.

### Synthesis of compounds

A cooled solution of 4-(chlorosulfonyl)benzoic acid (1100 mg, 5.0 mmol) in dichloromethane (DCM, 100 mL) was treated with cyclohexanamine (495 mg, 5.0 mmol) followed by triethylamine (707 mg, 7.0 mmol). The reaction mixture was permitted to warm to room temperature and stirred for 12 h. The reaction solution was poured into water and extracted with DCM. The combined organic layers were washed with water and brine and then dried over Na_2_SO_4_. The mixture was filtered and the filtrate was concentrated. The residue was purified by Combi-flash column, providing compound 4-(*N*-cyclohexylsulfamoyl)benzoic acid (300 mg, 21%). 4-(*N*-cyclohexylsulfamoyl)benzoic acid (103 mg, 0.36 mmol) was dissolved in water (5 mL) and treated with NaOH (14.6 mg, 0.36 mmol) at 0 °C. The resulting solution was stirred for 30 min, which was then lyophilized to give the desired product sodium 4-(*N*-cyclohexylsulfamoyl)benzoate (**40**, 100 mg, 91%). ^1^H NMR (400 MHz, DMSO-*d6*): δ 7.74 (d, *J* = 8.4 Hz, 2H), 7.41 (d, *J* = 6.8 Hz, 2H), 3.60–3.61 (m, 1H), 1.46–1.58 (m, 5H), 0.99–1.16 (m, 5H). LC-MS (ESI): *m*/*z* 282.1 (M-Na)^−^. All the other compounds are detailed in SI Materials and Methods.

### Animals

Mice (6–8 weeks old) were purchased from Jackson laboratory (Bar Harbor, ME) and housed in specific pathogen-free rooms within the animal-care facilities of the University of Pittsburgh Cancer Institute. All procedures of the mouse work were approved by the Institutional Animal Care and Use Committee at the University of Pittsburgh.

### *Ex vivo* HSC expansion

HSCs expansion medium consists of BIT9500 (Stem Cell Technologies) supplemented with 50 ng/mL recombinant mouse (rm) SCF, 20 ng/mL FMS-like tyrosine kinase 3 ligand (Flt3L) and 10 ng/mL thrombopoietin (Tpo) (all from PeproTech, Inc.). p18SMI compounds were added where indicated. Bone marrow cells were harvested from C57BL/6 mice and made into a single-cell suspension. After immunomagnetic c-Kit enrichment (cKit-conjugated microbeads [MiltenyiBiotec, Bergisch-Gladbach]), cells were washed and resuspended in HSCs expansion medium. After culture, total nucleated cell counts were obtained, and a fraction of mononuclear cells (MNCs) or whole bone marrow cells were stained for flow cytometry analysis. Frequency of each cell population was determined by independently analyzing more than 5 × 10^5^ cells per sample in triplicate.

### Flow cytometry

For stem-cell quantitation, the cultured bone marrow nucleated cells were co-stained with a combination of PE-Cy7 labeled antibodies against mouse CD3, CD4, CD8, B220, Gr-1, Mac-1 and TER-119, anti-Sca-1-PercpCy5.5, anti-CD48-PE and anti-CD150-APC (all from Biolegend). DAPI was used for dead-cell discrimination. Due to the presence of cytokine stem cell factor (SCF), we could not detect the c-Kit marker of those cultured cells. So hereafter we did not use c-Kit in detecting subpopulation. Flow cytometry and flow sorting were conducted with a CyAn (DakoCytomation) or a FACSCalibur system (BD Biosciences). FlowJo software Version 7.6.2 (TreeStar) was used for data analysis.

### Single cell culture *in vitro*

Single CD34^-^LKS cells from C57BL/6 mice were individually deposited into each well of a 96-well plate along with 100 μL of serum free medium (Gibco) containing 100 ng/mL of mouse SCF, 10 ng/mL of mouse IL-3, 25 ng/mL of human TPO, 2 U/mL of human EPO and p18SMI compounds (different dilutions). Cells were cultured in a 37 °C, 5% CO_2_ incubator for 14 days for *ex vivo* colony formation. Colonies were then cytospined and analyzed using flow cytometry. Neutrophils (n), macrophages (m), erythroblasts (E) or megakaryocytes (M) were identified based on their cell surface maker, Gr-1, Mac-1, Ter119 and CD41. Cells were measured on an FACS analyzer (FACSArray, BD Biosciences). Antibodies were used as following: anti-Gr-1-Percp-cy5.5, anti-Mac-1-APC-cy7, anti-Ter119-APC, anti-CD41-PE (BD Biosciences). The selection of cell surface marker of each subpopulation cells were considered based on the manufacture’s guide (BD Biosciences). To be specific, Gr-1 cell marker represents neutrophils (n), Mac-1 represents macrophages (m), Ter119 represents erythroblasts (E) and CD41 represents megakaryocytes (M). The wells from each group that generated all lineages (nmEM) were counted. The ratio of p18SMI compound treatment group to the control group (DMSO-treated) was calculated and fitted to a curve to calculate the ED_50_ value using GraphPad Prism v6.0.

### Cobblestone area forming cells (CAFC) assay and limiting dilution assay (LDA)

Freshly isolated bone marrow MNCs from mice, 6–8 weeks old, were plated in a 24-well plate with 1 mL Dexter’s medium at a density of 2 × 10^6^ cells per mL in the absence or presence of different doses of the tested compounds. The culture was maintained at 33 °C with 5% CO_2_. The number of cobblestone areas (CAs) was counted weekly. The medium was also replaced with half fresh medium on a weekly basis. For the limiting dilution analysis (LDA) of the test cells, different limiting doses were plated on the irradiated stromal cells in 96-well plates containing 200 μL of M5300 medium (StemCell Technologies) supplemented with 10^−6^ M hydrocortisone. Each group was done with 20 replicates well. Small-molecule compounds were added to the co-culture system at different doses. Half of the medium in each well was replaced with fresh medium containing the test compounds weekly. The existence of CA in each well was evaluated under an inverted microscope at weekly intervals for 5 weeks. Based on the Poisson distribution of the negative wells, the frequency of long-term initiating cells was calculated with L-Calc software (StemCell Technologies).

### Apoptosis analysis

c-Kit enriched BM cells were cultured for 5 days with cytokine combination plus compound 40 or DMSO. As positive controls, primary uncultured bone marrow cells were treated by ultraviolet radiation (UV) for 10 minutes prior to the staining process for apoptosis analysis. Apoptosis and cell death were measured by AnnexinV and DAPI staining according to the manufacturer’s instructions in the Annexin V-FITC Apoptosis Detection Kit (BD Biosciences). Apoptosis was measured on an FACS analyzer (LSR Fortessa, BD Biosciences). The data was analyzed using FlowJo software (Treestar, Inc.).

### CFSE assay

Prior to culture, cKit-enriched BM cells (1 × 10^6^/mL) were labeled with 5- (and 6-) carboxy-fluorescein diacetatesuccinimidylester (CFSE) dye (3 μM, Molecular Probes) in PBS supplemented with 0.1% bovine serum albumin. Labeling was performed in the dark at 37 °C for 10 min. Labeling was stopped by the addition of 5 volumes of ice cold PBS. 4 days after culture with cytokine plus compound, labeled cells were harvested and stained with the antibody cocktail for lineage markers, Sca-1, CD48, and CD150. During cell division, CFSE is distributed equally between daughter cells so that the generation of cells could be reflected by the content of CFSE and measured by the intensity of fluorescence. The CyAnsystem (DakoCytomation) was used for data acquisition. The data was analyzed using cells/Proliferation module of FlowJo software (Treestar, Inc.), which would fit a curve of the input data, automatically generate peaks standing for subpopulations with different fluorescence intensity and calculate the percentage of each peak and divided statistics results. The deailed gating strategy can be found in Supplemental Information.

### Human cord blood CD34^+^ cell culture and primary screen

Human cord blood samples were collected from consenting donors according to ethically approved procedures at Tianjin Central Hospital of Gynecology Obstetrics (Tianjin, China), under a protocol approved by the Ethical Committee on Medical Research at the Institute of Hematology. Human CD34^+^ cord blood cells were isolated using human CD34 MicroBead Kit (MiltenyiBiotec) according to the manufacturer’s protocol after settling red cells with HES and red cells lysis with ACK Lysis Buffer.

Human CD34^+^ cells were cultured in expansion medium consisting of StemSpan SFEM (StemCell Technologies) supplemented with 100 ng/mL human stem cell factor (SCF, PeproTech), 50 ng/mL human thrombopoietin (TPO, PeproTech), 100 ng/mL human FMS-like trysine kinase 3 ligand (Flt3L, PeproTech). Human CD34^+^ cells were resuspended in expansion medium (5 × 10^4^ cells/mL) before being aliquoted in 96-well plates (Costar, Corning Incorporated). Compounds, all with a final concentration of 200 nM, were dissolved in DMSO and added immediately in cell suspension after plating. The final concentration of DMSO in the control group was the same as in the compounds-treated group. Cells were cultured at 37 °C in 5% CO_2_. Based on the results of primary screening upon human cells and the results of ED_50_, we chose three compounds for further experiments. The expansion medium was changed to StemSpan SFEM (StemCell Technologies) supplemented with 100 ng/mL human stem cell factor (SCF, PeproTech), 100 ng/mL human thrombopoietin (TPO, PeproTech), 100 ng/mL human FMS-like trysine kinase 3 ligand (Flt3L, PeproTech) and 100 ng/mL human interleukin-6 (IL-6, PeproTech) for better expansion effect. The incubation condition was consistent with before.

Phenotypes analysis of cultured cells was performed on a BD FACS Array (Becton Dickinson Biosciences) using a combination of the following antibodies: APC anti-CD34 (BD Biosciences), PE anti-CD49f (BD Biosciences). Stained cells were washed once with PBS and then measured and analyzed on an FACS analyzer (FACSArray, BD Biosciences). The calculating formula is showed as following:


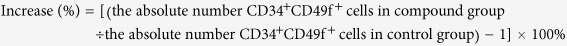


### MTT assay of top compounds in cell lines

To evaluate the toxicity of our top compounds, we performed a cell proliferation assay using 32D cells as well as leukemia cell lines and myeloma cell lines. Weihi-3 cells were cultured at 37 °C under 5% CO_2_ in RPMI 1640 medium supplemented with 10%FBS. After 48 h, the supernatant was collected and filtered. 32D cells were cultured in RPMI 1640 with 10% FBS and 10% supernatant collected from Weihi-3 cells. 8,000 32D cells or leukemia cell line (KG-1 or KG-1a) or myeloma cell line (MM.1S or RPMI 8226) were plated in a 96-well plate in 198 μL culture media per well, and then cultured (37 °C, 5% CO_2_) overnight. 2 μL of serially diluted compounds dissolved in saline or DMSO were added to each well and incubated (37 °C, 5% CO_2_) for 72 h. The concentration of DMSO was equal to the one in corresponding compound group. 20 μL of MTT solution was added to each well and then incubated (37 °C, 5% CO_2_) for 4 h. The percentage of cell survival was determined using the MTT assay, as previously described^39^,^40^. The data was analyzed using GraphPad Prism v6.0.

### Statistical analysis

An unpaired two-tailed Student’s *t*-test with assumption of experimental samples of equal variance was used for all statistical analyses.

## Additional Information

**How to cite this article**: Xie, X.-Q. *et al.* Discovery of novel INK4C small-molecule inhibitors to promote human and murine hematopoietic stem cell *ex vivo* expansion. *Sci. Rep.* **5**, 18115; doi: 10.1038/srep18115 (2015).

## Supplementary Material

Supplementary Information
