# Supplementary material for: Discovery of novel INK4C small-molecule inhibitors to promote human and murine hematopoietic stem cell ex vivo expansion
Source: Sci Rep. 2015 Dec 18;5:18115. doi: 10.1038/srep18115 (PMC4683533; doi:10.1038/srep18115)
Supplement: Supplementary Information [file srep18115-s1.pdf]

Supporting Information

Discovery of novel INK4C small-molecule inhibitors to promote human and murine hematopoietic stem cell *ex vivo* expansion

Xiang-Qun Xie[1,3,] Peng Yang[1,3], Yu Zhang[2,3], Peng Zhang[1], Liping Wang[1], Yahui Ding[2], Ming Yang[2], Qin Tong[1], Haizi Cheng[1], Qing Ji[2], Terence McGuire[1], Weiping Yuan[2], Tao Cheng[2,]* and Yingdai Gao[2,]**

[1]Department of Pharmaceutical Sciences and Computational Chemical Genomics Screening Center, School of Pharmacy; NIH National Center of Excellence for Computational Drug Abuse Research; Drug Discovery Institute; Department of Computational Biology and Structural Biology, School of Medicine, University of Pittsburgh, Pittsburgh, Pennsylvania 15260, United States.

[2]Key Laboratory of Experimental Hematology, Institute of Hematology and Blood Diseases Hospital, Chinese Academy of Medical Sciences and Peking Union Medical College, Tianjin 300020, P. R. China.

[3] These authors contributed equally to this work.

* Correspondence and requests for materials should be addressed:

Xiang-Qun Xie, e-mail: xix15@pitt.edu;

Tao Cheng, e-mail: chengtao@ihcams.ac.cn and

Yingdai Gao, e-mail: ydgao@ihcams.ac.cn

Methods

Chemistry. All reagents were purchased from commercial sources and used without further purification. Analytical thin-layer chromatography (TLC) was performed on SiO_2 plates on alumina. Visualization was accomplished by UV irradiation at 254 nm. Flash column chromatography was performed using the BiotageIsolera flash purification system with SiO_2 60 (particle size 0.040–0.055 mm, 230–400 mesh). ^{1}H NMR was recorded on a Bruker 400 MHz spectrometer. Splitting patterns are indicated as follows: s, singlet; d, doublet; t, triplet; m, multiplet; br, broad peak. Purity of all final derivatives for biological testing was confirmed to be > 95%, as determined by the following conditions: a Shimadzu HPLC instrument with a Hamilton reversed phase column (HxSil, C18, 3 μm, 2.1 mm × 50 mm (H2)); eluent A consisting of 5% CH_3CN in H_2O; eluent B consisting of 90% CH_3CN in H_2O; flow rate of 0.2 mL / min; UV detection, 254 and 214 nm.

General procedure for synthesis of lactone intermediate. To a mixture of an appropriate phenol and a *β*-keto ester in a 1:1.5 molar ratio was slowly added excess H_2SO_4 (98%) within 1 h at 0 ^oC. The mixture was stirred at room temperature until the reaction was complete, as monitored by TLC. The mixture was poured into ice-water and allowed to stand overnight. The precipitated solid was filtered, washed with water until neutral, and dried *in vacuo* to prepare the product[1].

Synthesis of 4-(acetylamine)-benzenesulfonyl chloride intermediate. 4.56 g (2.6 mL, 39.14 mmol) of chlorosulfonic acid was slowly added to 1.0 g (7.40 mmol) of *N*-phenyl-acetamide. The resulting mixture was stirred and heated at 60 ^oC for 30 min. After cooling, the 4-(acetylamine)-benzenesulfonyl chloride was filtered through a Buckner funnel, washed twice

with 50 mL of water, and recovered as a white hygroscopic powder in 85% yield. The product was used in the next step without further purification.

General procedure of coupling reaction between sulfonyl chlorides and substituted phenols. A cooled solution of 4-acetamidobenzene-1-sulfonyl chloride (466 mg, 2.0mmol) in THF (10 mL) was treated with 7-hydroxy-4-methyl-2H-chromen-2-one (352 mg, 2.0mmol) followed by triethylamine (243 mg, 2.4 mmol). The reaction mixture was permitted to warm to room temperature and stirred for 12 h. The reaction mixture was filtered and the filtrate was concentrated. The residue was purified by Combi-flash column, providing the desired product, XIE18-6 (356 mg, 48%).

General procedure of coupling reaction between sulfonyl chlorides and substituted amines. 4-Acetamidobenzene-1-sulfonyl chloride (466 mg, 2.0 mmol) and methyl (*R*)-2-amino-2-phenylacetate (403 mg, 2.0 mmol) were dissolved in 20 mL of water. The mixture was added with K_2CO_3 (414 mg, 3.0 mmol) and stirred at room temperature for 12 h. The precipitated solid was filtered, washed with water, and dried *in vacuo* to obtain the crude product, which was recrystallized in ethanol to prepare the final component, compound **22** (368 mg, 46%). ^{1}H NMR (400 MHz, DMSO-*d6*): 10.25 (s, 1H), 8.77 (s, 1H), 7.65-7.74 (m, 4H), 7.28-7.34 (m, 5H), 4.97 (s, 1H), 3.45 (s, 3H), 2.08 (s, 3H). HPLC-MS (ESI): *m*/*z* 363.0 (M+H) $^{+}$.

2-Oxo-2H-chromen-7-yl 4-acetamidobenzenesulfonate (**1**). Yield: 52%. ^{1}H NMR (400 MHz, DMSO-d_6) δ 10.51 (s, 1H), 8.05 (d, J = 9.6 Hz, 1H), 7.82–7.86 (m, 4H), 7.75 (d, J = 8.8 Hz, 1H), 7.12 (d, J = 2.4 Hz, 1H), 7.02-7.04 (m, 1H), 6.51 (d, J = 9.6 Hz, 1H), 2.11 (s, 3H). LC-MS (ESI): *m*/*z* 360.0 (M + H) $^{+}$.

4-Isopropyl-2-oxo-2H-chromen-7-yl 4-acetamidobenzenesulfonate (**2**). Yield: 81%. ^{1}H NMR (400 MHz, DMSO-*d6*): 10.51 (s, 1H), 7.93 (d, *J* = 8.8 Hz, 1H), 7.85 (s, 4H), 7.12 (d, *J* = 2.4 Hz, 1H), 7.04-7.07 (m, 1H), 6.35 (s, 1H), 3.30-3.32 (m, 1H), 2.11 (s, 3H), 1.24 (d, *J* = 6.8 Hz, 6H). HPLC-MS (ESI): *m*/*z* 402.1 (M+H) $^{+}$.

N-(4-(*N*-phenethylsulfamoyl)phenyl)acetamide (**3**). Yield: 65%. ^{1}H NMR (400 MHz, DMSO-d_6) δ 6.93 (d, *J* = 9.6 Hz, 4H), 6.31-6.46 (m, 5H), 2.28 (t, *J* = 7.6 Hz, 2H), 1.92 (t, *J* = 7.6 Hz, 2H), 1.36 (s, 3H). LC-MS (ESI): *m*/*z* 319.0 (M + H) $^{+}$.

N-(4-(*N*-(4-fluorophenethyl)sulfamoyl)phenyl)acetamide (**4**). Yield: 32%. ^{1}H NMR (400 MHz, DMSO-d_6) δ 6.94 (s, 4H), 6.32-6.35 (m, 2H), 6.14-6.18 (m, 2H), 2.27 (t, *J* = 7.2 Hz, 2H), 1.92 (t, *J* = 7.2 Hz, 2H), 1.36 (s, 3H). LC-MS (ESI): *m*/*z* 337.1 (M + H) $^{+}$.

N-(4-(*N*-benzylsulfamoyl)phenyl)acetamide (**5**). Yield: 55%. ^{1}H NMR (400 MHz, DMSO-d_6) δ 10.30 (s, 1H), 8.00 (t, *J* = 6.4 Hz, 1H), 7.72-7.77 (m, 4H), 7.21-7.31 (m, 5H), 3.96 (d, *J* = 6.0 Hz, 2H), 2.10 (s, 3H). LC-MS (ESI): *m*/*z* 305.2 (M + H) $^{+}$.

N-(4-(*N*-(3,4-dichlorobenzyl)sulfamoyl)phenyl)acetamide (**6**). Yield: 32%. ^{1}H NMR (400 MHz, DMSO-d_6) δ 10.30 (s, 1H), 8.12 (t, *J* = 6.4 Hz, 1H), 7.68-7.75 (m, 4H), 7.23-7.55 (m, 3H), 3.99 (d, *J* = 6.4 Hz, 2H), 2.09 (s, 3H). LC-MS (ESI): *m*/*z* 372.9 (M + H) $^{+}$.

N-(4-(*N*-(4-(diethylamino)benzyl)sulfamoyl)phenyl)acetamide (**7**). Yield: 60%. ^{1}H NMR (400 MHz, DMSO-d_6) δ 10.28 (s, 1H), 7.69-7.75 (m, 5H), 6.97 (d, *J* = 8.4 Hz, 2H), 6.52 (d, *J* = 8.4 Hz, 2H), 3.79 (d, *J* = 6.0 Hz, 2H), 3.28-3.35 (m, 4H), 2.09 (s, 3H), 1.05 (t, *J* = 6.8 Hz, 6H). LC-MS (ESI): *m*/*z* 376.1 (M + H) $^{+}$.

N-(4-(*N*-(4-bromophenyl)sulfamoyl)phenyl)acetamide (**8**). Yield: 71%. ^{1}H NMR (400 MHz, DMSO-d_6) δ 10.28 (s, 1H), 7.67-7.73 (m, 4H), 7.46 (d, J = 8.4 Hz, 2H), 7.21 (d, J = 8.8 Hz, 2H), 3.91 (s, 2H), 2.09 (s, 3H). LC-MS (ESI): m/z 382.8 (M + H) $^{+}$.

N-(4-(*N*-phenylsulfamoyl)phenyl)acetamide (**9**). Yield: 53%. ^{1}H NMR (400 MHz, DMSO-d_6) δ 10.28 (s, 1H), 10.14 (s, 1H), 7.66-7.71 (m, 4H), 6.99-7.24 (m, 5H), 2.06 (s, 3H). LC-MS (ESI): m/z 291.1 (M + H) $^{+}$.

N-(4-(*N*-(p-tolyl)sulfamoyl)phenyl)acetamide (**10**). Yield: 47%. ^{1}H NMR (400 MHz, DMSO-d_6) δ 10.27 (s, 1H), 9.96 (s, 1H), 7.63-7.69 (m, 4H), 7.02 (d, J = 8.4 Hz, 2H), 6.95 (d, J = 8.4 Hz, 2H), 2.18 (s, 3H), 2.06 (s, 3H). LC-MS (ESI): m/z 305.2 (M + H) $^{+}$.

N-(4-(*N*-(o-tolyl)sulfamoyl)phenyl)acetamide (**11**). Yield: 15%. ^{1}H NMR (400 MHz, DMSO-d_6) δ 10.27 (s, 1H), 9.97 (s, 1H), 7.63-7.69 (m, 4H), 7.02 (d, J = 8.4 Hz, 2H), 6.95 (d, J = 8.4 Hz, 2H), 2.18 (s, 3H), 2.06 (s, 3H). LC-MS (ESI): m/z 305.1 (M + H) $^{+}$.

N-(4-(*N*-(4-chlorophenyl)sulfamoyl)phenyl)acetamide (**12**). Yield: 3.0%. ^{1}H NMR (400 MHz, DMSO-d_6) δ 10.31 (s, 1H), 10.30 (s, 1H), 7.66-7.72 (m, 4H), 7.28 (d, J = 8.4 Hz, 2H), 7.08 (d, J = 8.8 Hz, 2H), 2.06 (s, 3H). LC-MS (ESI): m/z 325.1 (M + H) $^{+}$.

N-(4-(*N*-(4-(dimethylamino)phenyl)sulfamoyl)phenyl)acetamide (**13**). Yield: 35%. ^{1}H NMR (400 MHz, DMSO-d_6) δ 10.27 (s, 1H), 9.52 (s, 1H), 7.67 (d, J = 8.8 Hz, 2H), 7.57 (d, J = 8.8 Hz, 2H), 6.85 (d, J = 8.8 Hz, 2H), 6.60 (d, J = 8.8 Hz, 2H), 2.82 (s, 6H), 2.07 (s, 3H). LC-MS (ESI): m/z 334.1 (M + H) $^{+}$.

N-(4-(*N*-(2-fluoro-5-(trifluoromethyl)phenyl)sulfamoyl)phenyl)acetamide (**14**). Yield: 17%. ^{1}H NMR (400 MHz, MeOD) δ 7.77-7.89 (m, 5H), 7.32-7.48 (m, 2H), 2.21 (s, 3H). LC-MS (ESI): *m*/*z* 375.1 (M - H) $^{-}$.

N-(4-(*N*-(bicyclo[2.2.1]heptan-2-yl)sulfamoyl)phenyl)acetamide (**15**). Yield: 63%. ^{1}H NMR (400 MHz, DMSO-d_6) δ 10.29 (s, 1H), 7.70-7.77 (m, 4H), 7.29 (d, *J* = 7.2 Hz, 1H), 2.91-2.96 (m, 1H), 2.09 (s, 3H), 1.92-1.93 (m, 1H), 0.99-1.45 (m, 9H). LC-MS (ESI): *m*/*z* 309.0 (M + H) $^{+}$.

N-(4-(*N*-(cyclohexylmethyl)sulfamoyl)phenyl)acetamide (**16**). Yield: 38%. ^{1}H NMR (400 MHz, DMSO-d_6) δ 6.94-6.99 (m, 4H), 1.85-1.86 (m, 2H), 1.36 (s, 3H), 0.90-0.92 (m, 5H), 0.34-0.58 (m, 6H). LC-MS (ESI): *m*/*z* 311.2 (M + H) $^{+}$.

N-(4-(morpholinosulfonyl)phenyl)acetamide (**17**). Yield: 33%. ^{1}H NMR (400 MHz, DMSO-d_6) δ 10.40 (s, 1H), 7.83-7.85 (m, 2H), 7.67-7.69 (m, 2H), 3.63 (t, *J* = 4.8 Hz, 4H), 2.84 (t, *J* = 4.8 Hz, 4H), 2.11 (s, 3H). LC-MS (ESI): *m*/*z* 285.0 (M + H) $^{+}$.

N-(4-(*N*-cyclohexylsulfamoyl)phenyl)acetamide (**18**). Yield: 45%. ^{1}H NMR (400 MHz, DMSO-d_6) δ 10.29 (s, 1H), 7.71-7.76 (m, 4H), 7.49 (d, *J* = 7.2 Hz, 1H), 2.88-2.89 (m, 1H), 2.09 (s, 3H), 1.43-1.56 (m, 5H), 1.03-1.12 (m, 5H). LC-MS (ESI): *m*/*z* 297.2 (M + H) $^{+}$.

N-(4-(*N*-(piperidin-1-yl)sulfamoyl)phenyl)acetamide (**19**). Yield: 36%. ^{1}H NMR (400 MHz, DMSO-d_6) δ 7.04-7.06 (m, 2H), 6.95-6.97 (m, 2H), 1.71 (t, *J* = 5.2 Hz, 4H), 1.38 (s, 3H), 0.65-0.71 (m, 4H), 0.50-0.52 (m, 2H). LC-MS (ESI): *m*/*z* 298.0 (M + H) $^{+}$.

N-(4-(*N*-cyclopentylsulfamoyl)phenyl)acetamide (**20**). Yield: 57%. ^{1}H NMR (400 MHz, DMSO-d_6) δ 10.35 (s, 1H), 7.80-7.82 (m, 2H), 7.26-7.75 (m, 2H), 3.11 (t, *J* = 6.4 Hz, 4H), 2.10 (s, 3H), 1.62-1.65 (m, 4H). LC-MS (ESI): *m*/*z* 269.0 (M + H) $^{+}$.

N-(4-(*N*-(2-hydroxyethyl)sulfamoyl)phenyl)acetamide (**21**). Yield: 5.8%. ^{1}H NMR (400 MHz, DMSO-d_6) δ 10.30 (bs, 1H), 7.69-7.76 (m, 4H), 3.35 (t, *J* = 6.4 Hz, 2H), 2.76 (t, *J* = 6.4 Hz, 2H), 2.09 (s, 3H). LC-MS (ESI): *m*/*z* 259.1 (M + H) $^{+}$.

Methyl (*S*)-2-((4-acetamidophenyl)sulfonamido)-2-phenylacetate (**23**). Yield: 52%. ^{1}H NMR (400 MHz, DMSO-*d6*): δ 10.27 (s, 1H), 8.77 (d, *J* = 9.6 Hz, 1H), 7.64-7.69 (m, 4H), 7.26-7.29 (m, 5H), 4.99 (d, *J* = 9.6 Hz, 1H), 3.46 (s, 3H), 2.08 (s, 3H). HPLC-MS (ESI): *m*/*z* 363.0 (M+H) $^{+}$.

4-Isopropyl-2-oxo-2H-chromen-7-yl butane-1-sulfonate (**24**). Yield: 34%. ^{1}H NMR (400 MHz, DMSO-*d6*): δ 8.02 (d, *J* = 8.8 Hz, 1H), 7.46 (d, *J* = 2.4 Hz, 1H), 7.34-7.37 (m, 1H), 6.38 (s, 1H), 3.63 (t, *J* = 7.6 Hz, 2H), 3.37-3.41 (m, 1H), 1.79-1.86 (m, 2H), 1.45-1.49 (m, 2H), 1.27 (d, *J* = 6.8 Hz, 6H), 0.94-0.95 (m, 3H). HPLC-MS (ESI): *m*/*z* 325.0 (M+H) $^{+}$.

4-Isopropyl-2-oxo-2H-chromen-7-yl phenylmethanesulfonate (**25**). Yield: 17%. ^{1}H NMR (400 MHz, DMSO-*d6*): δ 7.99 (d, *J* = 9.2 Hz, 1H), 7.43-7.52 (m, 5H), 7.32 (d, *J* = 2.4 Hz, 1H), 7.24-7.27 (m, 1H), 6.38 (s, 1H), 5.09 (s, 2H), 3.37-3.41 (m, 1H), 1.27 (d, *J* = 6.8 Hz, 6H). HPLC-MS (ESI): *m*/*z* 359.0 (M+H) $^{+}$.

4-Isopropyl-2-oxo-2H-chromen-7-yl 4-fluorobenzenesulfonate (**26**). Yield: 25%. ^{1}H NMR (400 MHz, DMSO-*d6*): δ 8.01-8.05 (m, 2H), 7.95 (d, *J* = 8.8 Hz, 1H), 7.53-7.57 (m, 2H), 7.21 (d, *J* = 2.4 Hz, 1H), 7.08-7.10 (m, 1H), 6.37 (s, 1H), 3.29-3.39 (m, 1H), 1.24 (d, *J* = 6.8 Hz, 6H). HPLC-MS (ESI): *m*/*z* 363.0 (M+H) $^{+}$.

4-Isopropyl-2-oxo-2H-chromen-7-yl 4-methylbenzenesulfonate (**27**). Yield: 36%. ^{1}H NMR (400 MHz, DMSO-*d6*): δ 7.93 (d, *J* = 8.8 Hz, 1H), 7.80-7.83 (m, 2H), 7.47-7.52 (m, 2H), 7.06-7.15

(m, 2H), 6.35 (s, 1H), 3.30-3.37 (m, 1H), 2.44 (s, 3H), 1.23 (d, *J* = 6.8 Hz, 6H). HPLC-MS (ESI): *m*/*z* 358.9 (M+H) $^+$.

4-Isopropyl-2-oxo-2H-chromen-7-yl 4-methoxybenzenesulfonate (**28**). Yield: 43%. ^{1}H NMR (400 MHz, DMSO-*d6*): δ 7.93 (d, *J* = 8.8 Hz, 1H), 7.84-7.87 (m, 2H), 7.18-7.21 (m, 2H), 7.05-7.14 (m, 2H), 6.35 (s, 1H), 3.88 (s, 3H), 3.31-3.37 (m, 1H), 1.23 (d, *J* = 6.8 Hz, 6H). HPLC-MS (ESI): *m*/*z* 375.0 (M+H) $^+$.

4-Isopropyl-2-oxo-2H-chromen-7-yl 4-chlorobenzenesulfonate (**29**). Yield: 40%. ^{1}H NMR (400 MHz, DMSO-*d6*): δ 7.93-7.96 (m, 3H), 7.77-7.80 (m, 2H), 7.22 (d, *J* = 2.4 Hz, 1H), 7.08-7.11 (m, 1H), 6.37 (s, 1H), 3.33-3.36 (m, 1H), 1.24 (d, *J* = 6.8 Hz, 6H). HPLC-MS (ESI): *m*/*z* 378.9 (M+H) $^+$.

4-Isopropyl-2-oxo-2H-chromen-7-yl 4-isopropylbenzenesulfonate (**30**). Yield: 35%. ^{1}H NMR (400 MHz, DMSO-*d6*): δ 7.94 (d, *J* = 8.8 Hz, 1H), 7.85-7.87 (m, 2H), 7.57-7.59 (m, 2H), 7.14 (d, *J* = 2.8 Hz, 1H), 7.08-7.11 (m, 1H), 6.35 (s, 1H), 3.31-3.37 (m, 1H), 3.01-3.08 (m, 1H), 1.22-1.24 (m, 12H). HPLC-MS (ESI): *m*/*z* 387.0 (M+H) $^+$.

4-(((4-Isopropyl-2-oxo-2H-chromen-7-yl)oxy)sulfonyl)benzoic acid (**31**). Yield: 21%. ^{1}H NMR (400 MHz, DMSO-*d6*): δ 8.12-8.14 (m, 2H), 8.01 (d, *J* = 8.8 Hz, 1H), 7.81-7.84 (m, 2H), 7.51 (d, *J* = 2.4 Hz, 1H), 7.38-7.41 (m, 1H), 6.36 (s, 1H), 3.40-3.45 (m, 1H), 1.29 (d, *J* = 6.8 Hz, 6H). HPLC-MS (ESI): *m*/*z* 389.0 (M+H) $^+$.

N-cyclohexyl-1-phenylmethanesulfonamide (**32**). Yield: 40%. ^{1}H NMR (400 MHz, DMSO-*d6*): δ 7.32-7.40 (m, 5H), 7.03 (s, 1H), 4.29 (s, 2H), 2.98-2.99 (m, 1H), 1.49-1.82 (m, 5H), 1.06-1.24 (m, 5H). HPLC-MS (ESI): *m*/*z* 254.2 (M+H) $^+$.

N-cyclohexyl-4-fluorobenzenesulfonamide (**33**). Yield: 33%. ^{1}H NMR (400 MHz, DMSO-*d6*): δ 7.85-7.87 (m, 2H), 7.68 (s, 1H), 7.39-7.45 (m, 2H), 2.92-2.95 (m, 1H), 1.43-1.58 (m, 5H), 1.02-1.19 (m, 5H). HPLC-MS (ESI): *m*/*z* 258.0 (M+H) $^{+}$.

N-cyclohexyl-4-methylbenzenesulfonamide (**34**). Yield: 44%. ^{1}H NMR (400 MHz, DMSO-*d6*): δ 7.69 (d, *J* = 8.4 Hz, 2H), 7.54 (s, 1H), 7.38 (d, *J* = 8.0 Hz, 2H), 2.89-2.92 (m, 1H), 2.39 (s, 3H), 1.43-1.58 (m, 5H), 1.01-1.16 (m, 5H). HPLC-MS (ESI): *m*/*z* 253.9 (M+H) $^{+}$.

4-Chloro-*N*-cyclohexylbenzenesulfonamide (**35**). Yield: 64%. ^{1}H NMR (400 MHz, DMSO-*d6*): δ 7.80-7.81 (m, 2H), 7.74 (s, 1H), 7.64-7.68 (m,2H), 2.93-2.97 (m, 1H), 1.44-1.58 (m, 5H), 1.04-1.19 (m, 5H). HPLC-MS (ESI): *m*/*z* 274.0 (M+H) $^{+}$.

N-cyclohexyl-4-methoxybenzenesulfonamide (**36**). Yield: 33%. ^{1}H NMR (400 MHz, DMSO-*d6*): δ 7.72-7.76 (m, 2H), 7.46 (d, *J* = 7.2 Hz, 1H), 7.08-7.12 (m,2H), 3.84 (s, 3H), 2.87-2.88 (m, 1H), 1.43-1.58 (m, 5H), 1.08-1.20 (m, 5H). HPLC-MS (ESI): *m*/*z* 270.0 (M+H) $^{+}$.

N-cyclohexyl-4-isopropylbenzenesulfonamide (**37**). Yield: 36%. ^{1}H NMR (400 MHz, DMSO-*d6*): δ 7.73 (d, *J* = 8.0 Hz, 2H), 7.55 (d, *J* = 7.2 Hz, 1H), 7.45 (d, *J* = 8.0 Hz, 2H), 2.93-3.01 (m, 2H), 1.36-1.58 (m, 5H), 1.23 (d, *J* = 6.8 Hz, 6H), 1.04-1.18 (m, 5H). HPLC-MS (ESI): *m*/*z* 282.0 (M+H) $^{+}$.

N-cyclohexyl-4-(dimethylamino)benzenesulfonamide (**38**). Yield: 6.8%. ^{1}H NMR (400 MHz, DMSO-*d6*): δ 7.74 (d, *J* = 9.2 Hz, 2H), 6.68 (d, *J* = 8.8 Hz, 2H), 2.97 (s, 6H), 2.63-2.68 (m, 1H), 1.54-1.79 (m, 5H), 1.06-1.27 (m, 5H). HPLC-MS (ESI): *m*/*z* 283.1 (M+H) $^{+}$.

4-(*N*-cyclohexylsulfamoyl)benzoic acid (**39**). Yield: 65%. ^{1}H NMR (400 MHz, DMSO-*d6*): δ 7.94 (d, *J* = 8.4 Hz, 2H), 7.68 (d, *J* = 8.4 Hz, 2H), 7.51 (bs, 1H), 2.90-2.91 (m, 1H), 1.42-1.55 (m, 5H), 0.99-1.11 (m, 5H). HPLC-MS (ESI): *m*/*z* 284.0 $(M+H)^{+}$.

4-Isopropyl-2-oxo-2H-chromen-7-yl 4-acetamidobenzoate (**41**). Yield: 81%. ^{1}H NMR (400 MHz, DMSO-*d6*): δ 10.41 (s, 1H), 8.11 (d, *J* = 8.8 Hz, 2H), 8.00 (d, *J* = 8.4 Hz, 1H), 7.82 (d, *J* = 8.8 Hz, 2H), 7.47 (d, *J* = 2.4 Hz, 1H), 7.34-7.36 (m, 1H), 6.36 (s, 1H), 3.41-3.46 (m, 1H), 2.12 (s, 3H), 1.29 (d, *J* = 6.8 Hz, 6H). HPLC-MS (ESI): *m*/*z* 366.0 $(M+H)^{+}$.

CFSE assay. Prior to culture, cKit-enriched BM cells (1×10^6/mL) were labeled with 5- (and 6-) carboxy-fluorescein diacetatesuccinimidylester (CFSE) dye (3 μM, Molecular Probes) in PBS supplemented with 0.1% bovine serum albumin. Labeling was performed in the dark at 37 °C for 10 min. Labeling was stopped by the addition of 5 volumes of ice cold PBS. 4 days after culture with cytokine plus compound, labeled cells were harvested and stained with the antibody cocktail for lineage markers, Sca-1, CD48, and CD150. During cell division, CFSE is distributed equally between daughter cells so that the generation of cells could be reflected by the content of CFSE and measured by the intensity of fluorescence. The CyAnsystem (DakoCytomation) was used for data acquisition. The data was analyzed using cells/Proliferation module of FlowJo software (Treestar, Inc.), which would fit a curve of the input data, automatically generate peaks standing for subpopulations with different fluorescence intensity and calculate the percentage of each peak and divided statistics results. Each peak represents one generation of cells. The original cells have the most intense fluorescence signal and emerge in the rightmost side of the X-axis. Upon cell division, the CFSE divides between daughter cells and the peak shifts left. While the Y-axis represents cell counts, the peak height represents the number of cells in each

generation. For our results, the four peaks represent four cell generations (first, second, third and fourth cell generations) going from the right to the left of the X-axis.

References:

1. Xie, L., Takeuchi, Y., Cosentino, L.M., McPhail, A.T. & Lee, K.H. Anti-AIDS agents. 42. Synthesis and anti-HIV activity of disubstituted (3'R,4'R)-3',4'-di-O-(S)-camphanoyl-(+)-cis-khellactone analogues. *J Med Chem* **44**, 664-71 (2001).

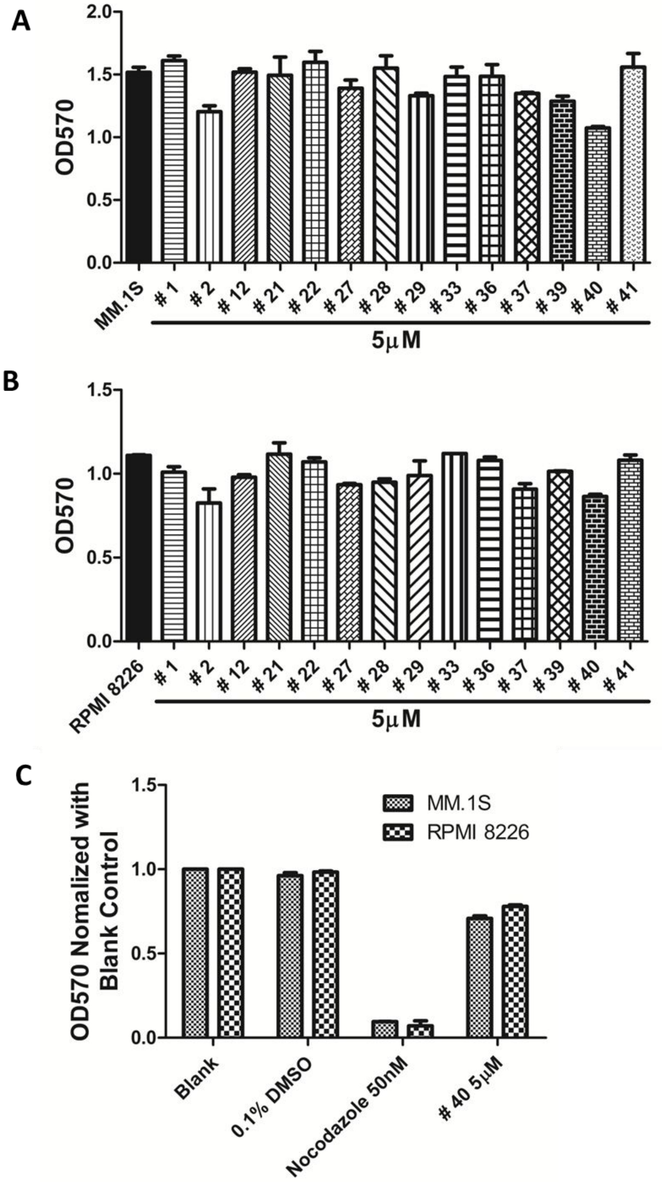

Figure S1. Cytotoxic effect of top compounds on myeloma cell lines. (A, B) MM.1S cells and RPMI 8226 cells (2×10^4 cells/well) were plated on 96-well plates. Cells were incubated with the indicated doses of compounds for 48 h. The percentage of cell survival was determined using the MTT assay. The data are the mean ± SEM of all experiments conducted in triplicate. (C) Cytotoxic effect of compound **40** and positive control Nocodazole on myeloma cell lines.

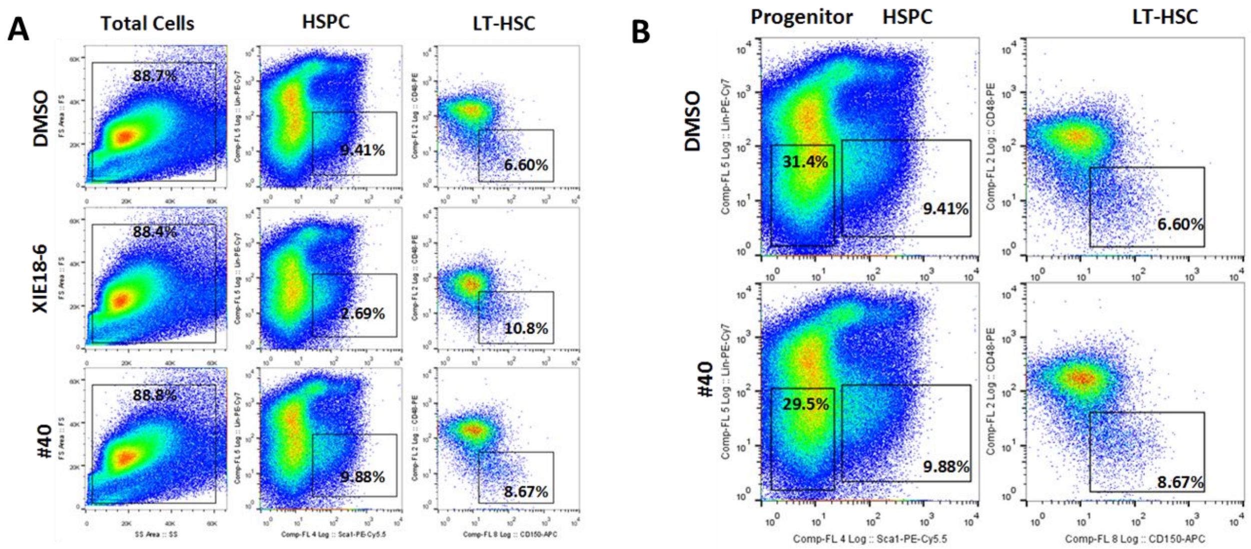

Figure S2.

Separation of hematopoietic stem / progenitor populations by flow cytometry. Gating scheme for the progenitor cells (Lin$^-$Sca1$^-$), HSPCs (Lin$^-$Sca1$^+$), and LT-HSCs (Lin$^-$Sca1$^+$CD48$^-$CD150$^+$) populations.

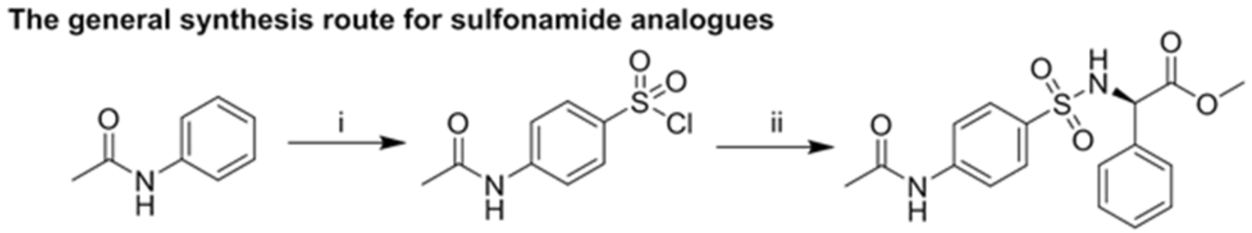

Figure S3. The general chemistry synthesis route for sulfonamide analogues. Reagents and conditions are as follows: i) HSO_3Cl, 60 °C; ii) K_2CO_3, H_2O, r.t, 12.

A

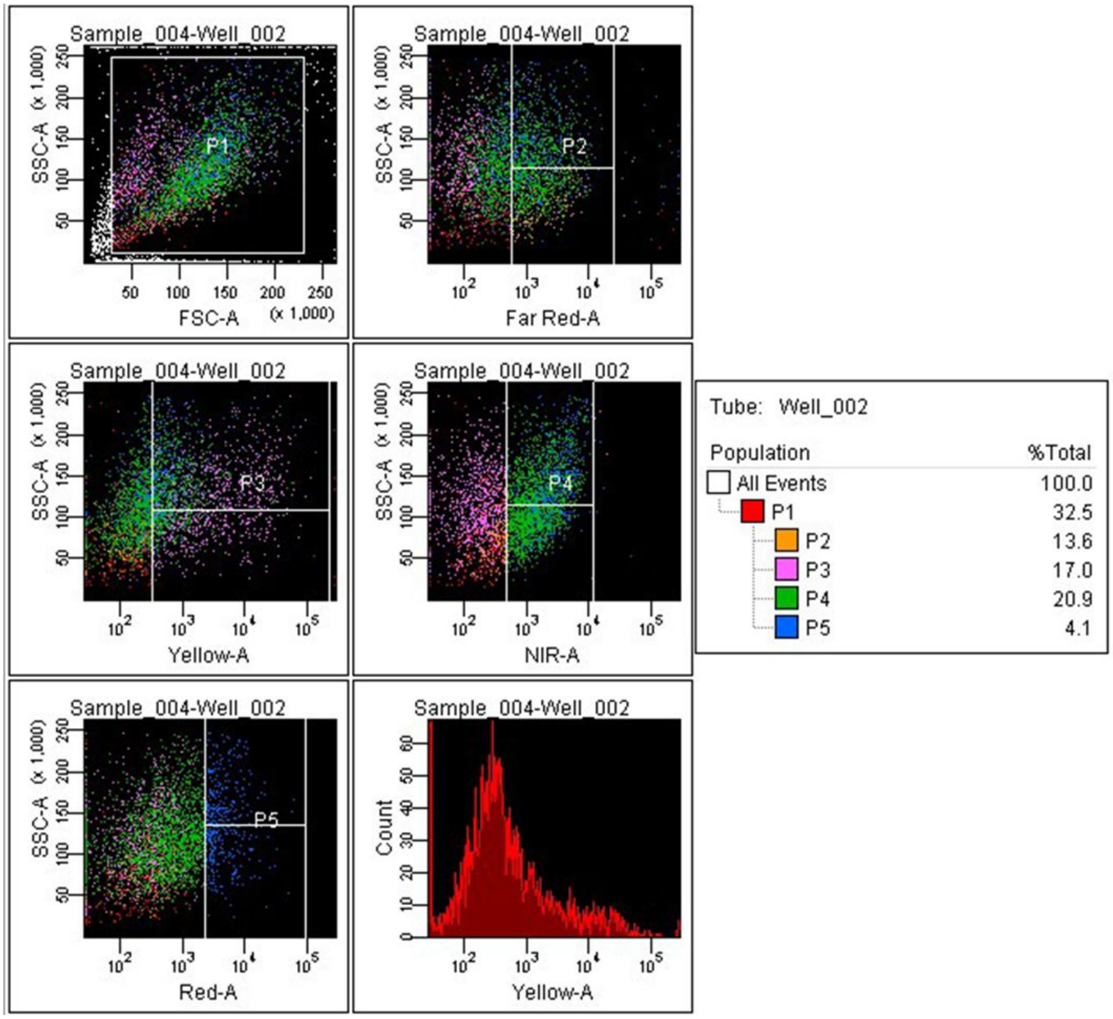
Sample_004-Well_002
SSC-A (x 1,000)
FSC-A
(x 1,000)
P1
Far Red-A
P2
Yellow-A
P3
NIR-A
P4
Red-A
P5
Count
Tube: Well_002
Population
%Total
All Events
100.0
P1
32.5
P2
13.6
P3
17.0
P4
20.9
P5
4.1

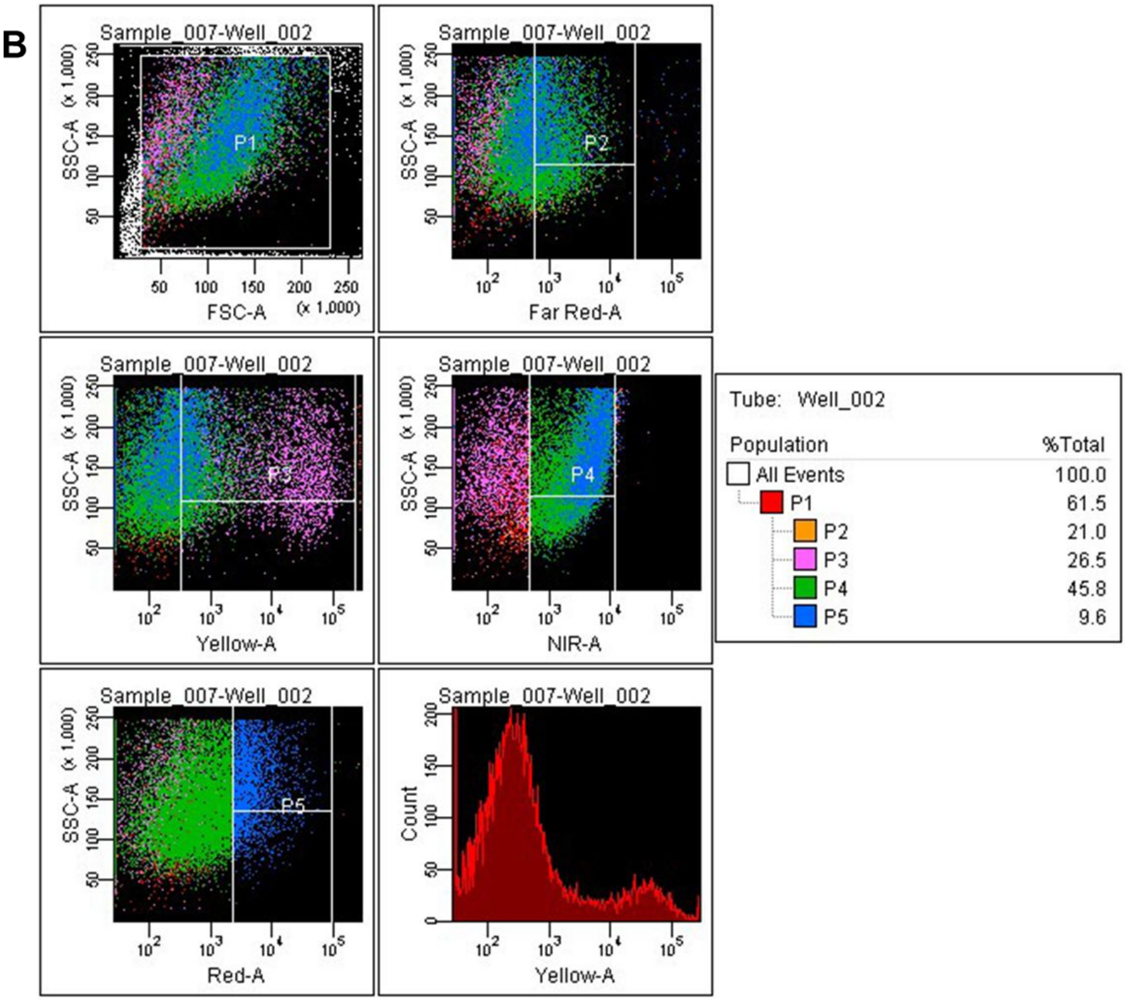
B
Sample_007-Well_002
SSC-A (x 1,000)
FSC-A
(x 1,000)
P1
Far Red-A
P2
Yellow-A
P3
NIR-A
P4
Red-A
P5
Count
Tube: Well_002
Population %Total
All Events 100.0
P1 61.5
P2 21.0
P3 26.5
P4 45.8
P5 9.6

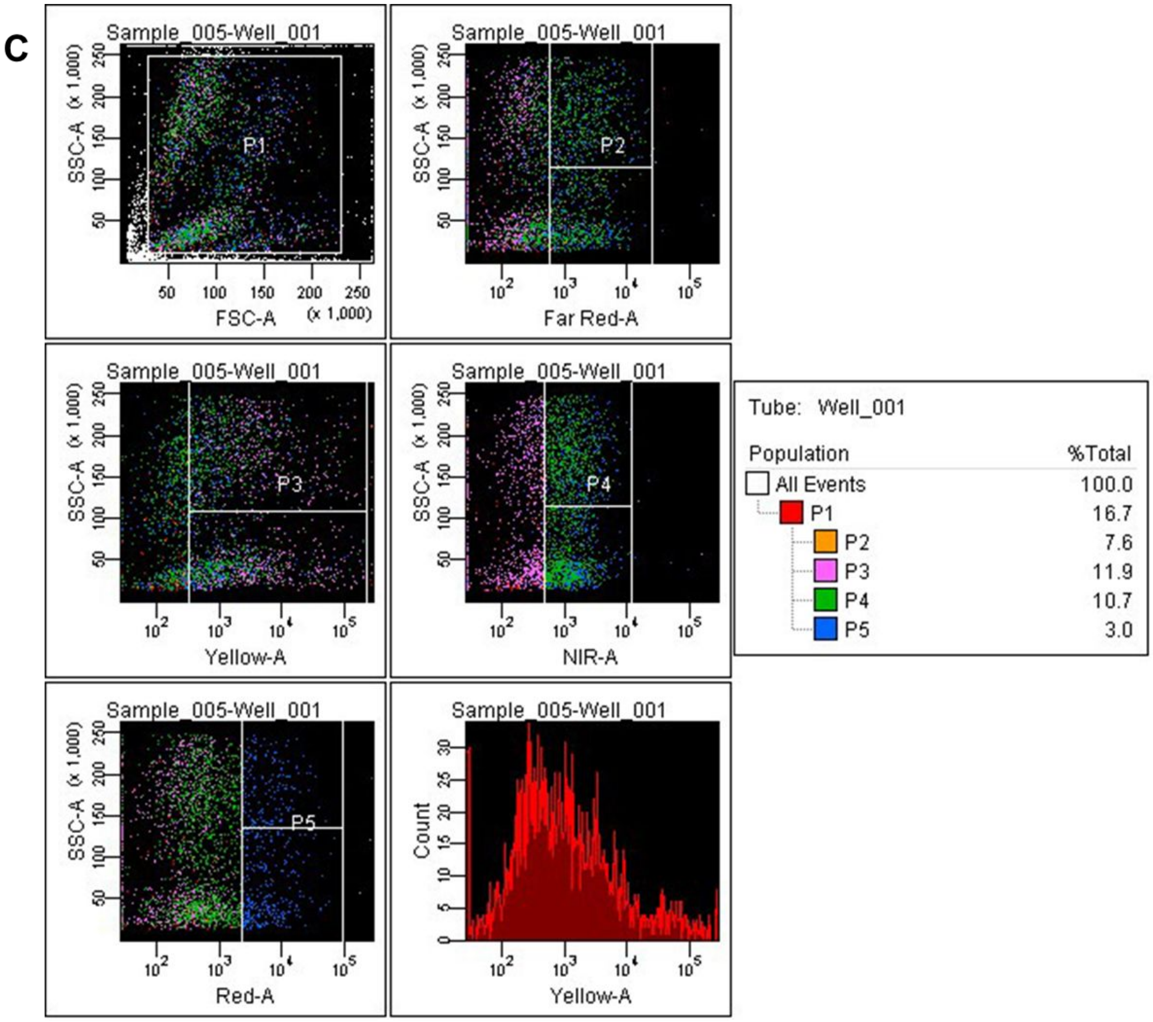

Figure S4. Representative flow cytometry plot figures of single cell colony assay in XIE18-6 (**A**), compound **40** (**B**) and control (**C**) groups, respectively. The matchup of parameters for each laser and fluorescence dye is as following: Far Red-Percp-cy5.5-Gr-1, Near Infrared (NIR) - APC-cy7-Mac-1, Red-APC-Ter119, Yellow-PE-CD41. Four lineage-positive wells were determined by the percentage of P2, P3 and P4 being greater than 5% and the percentage of P5 being greater than 3%.

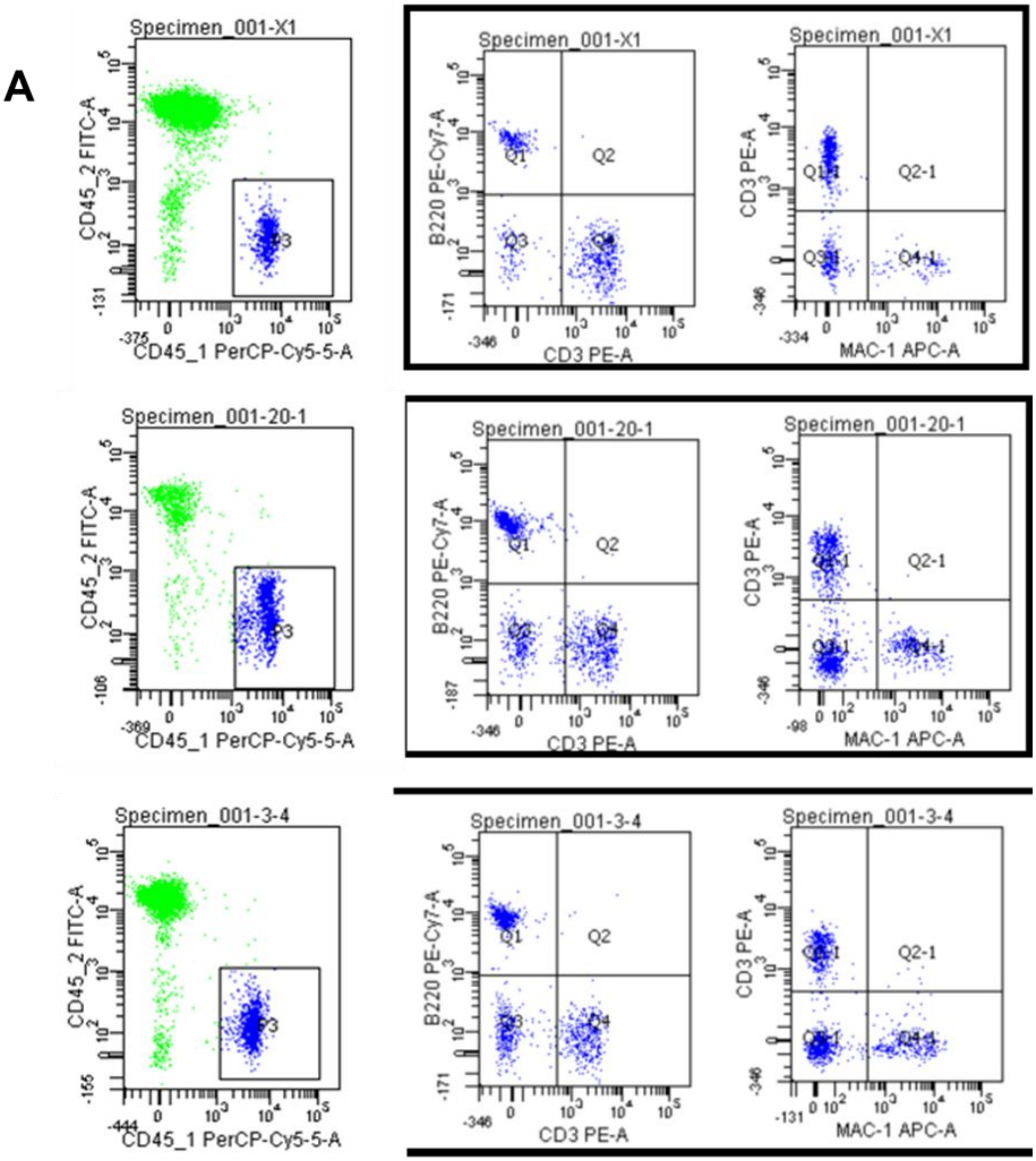
A
Specimen_001-X1
CD45_2 FITC-A
CD45_1 PerCP-Cy5-5-A
P3
B220 PE-Cy7-A
CD3 PE-A
Q1
Q2
Q3
Q4
Q1-1
Q2-1
Q3-1
Q4-1
MAC-1 APC-A
Specimen_001-20-1
Specimen_001-3-4

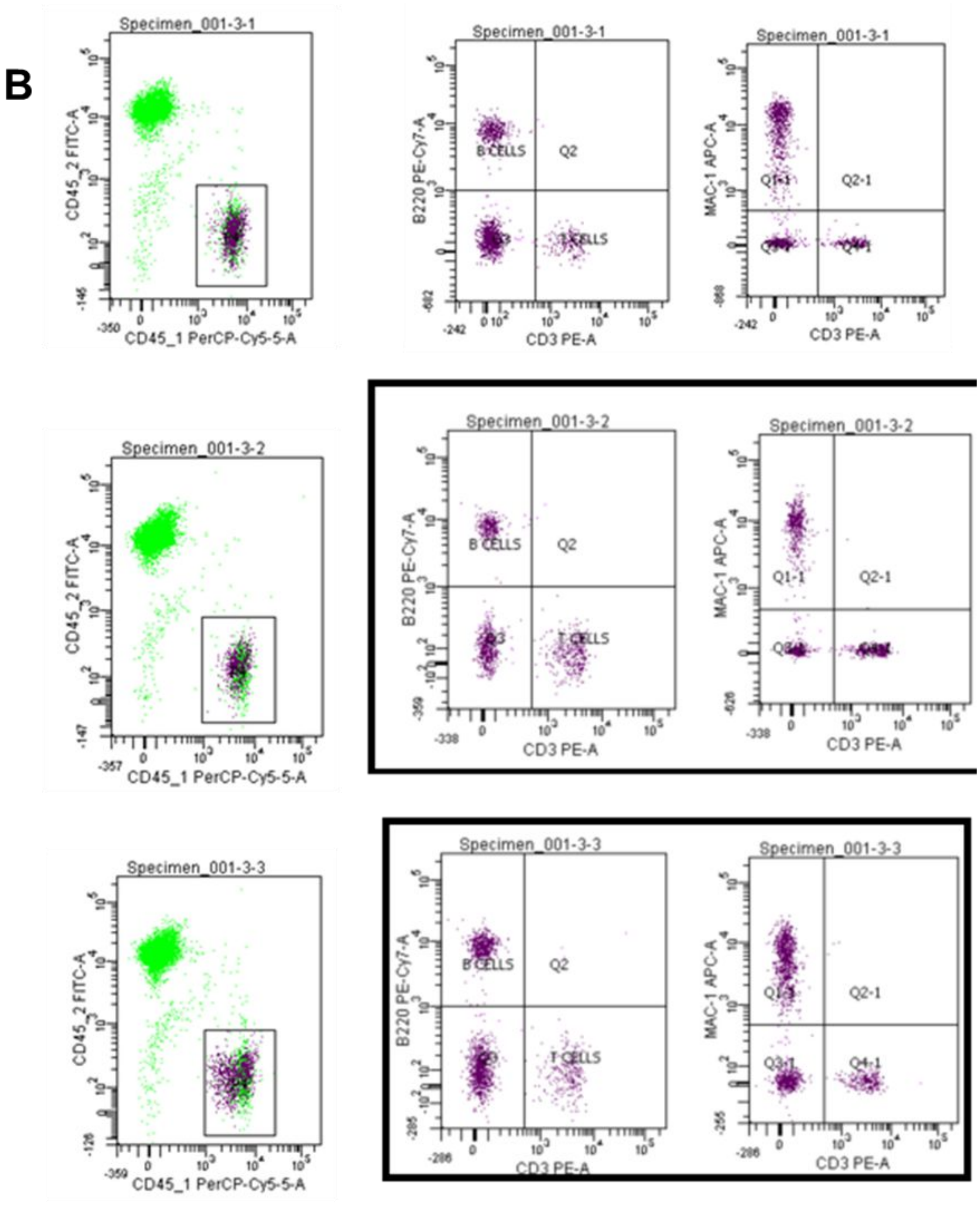
B
Specimen_001-3-1
Specimen_001-3-2
Specimen_001-3-3
CD45_2 FITC-A
CD45_1 PerCP-Cy5-5-A
B220 PE-Cy7-A
MAC-1 APC-A
CD3 PE-A
B CELLS
T CELLS
Q2
Q1-1
Q2-1
Q4-1

C

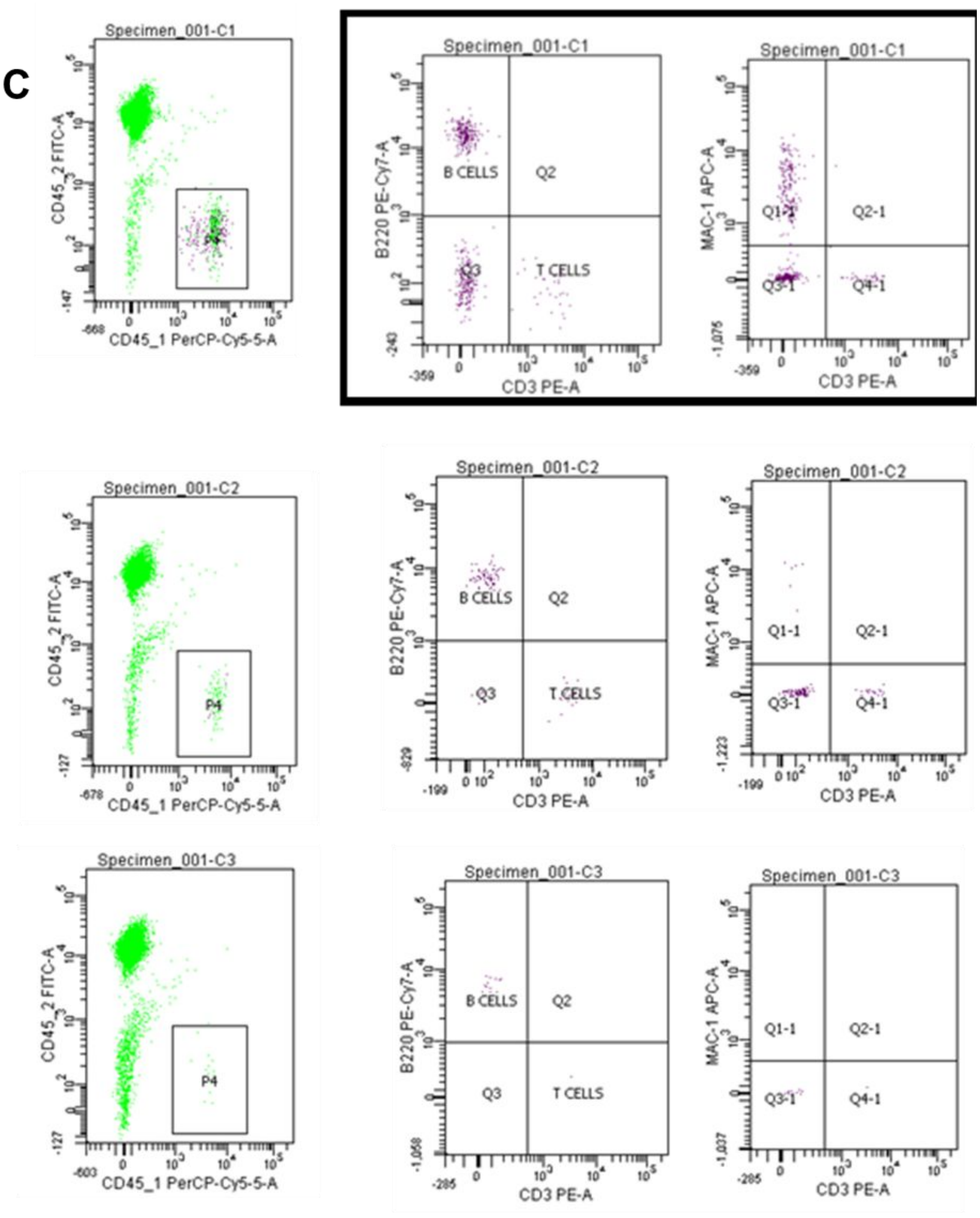
Specimen_001-C1
CD45_2 FITC-A
CD45_1 PerCP-Cy5-5-A
B220 PE-Cy7-A
CD3 PE-A
B CELLS
Q2
Q3
T CELLS
MAC-1 APC-A
Q1-1
Q2-1
Q3-1
Q4-1
Specimen_001-C2
P4
Specimen_001-C3

263

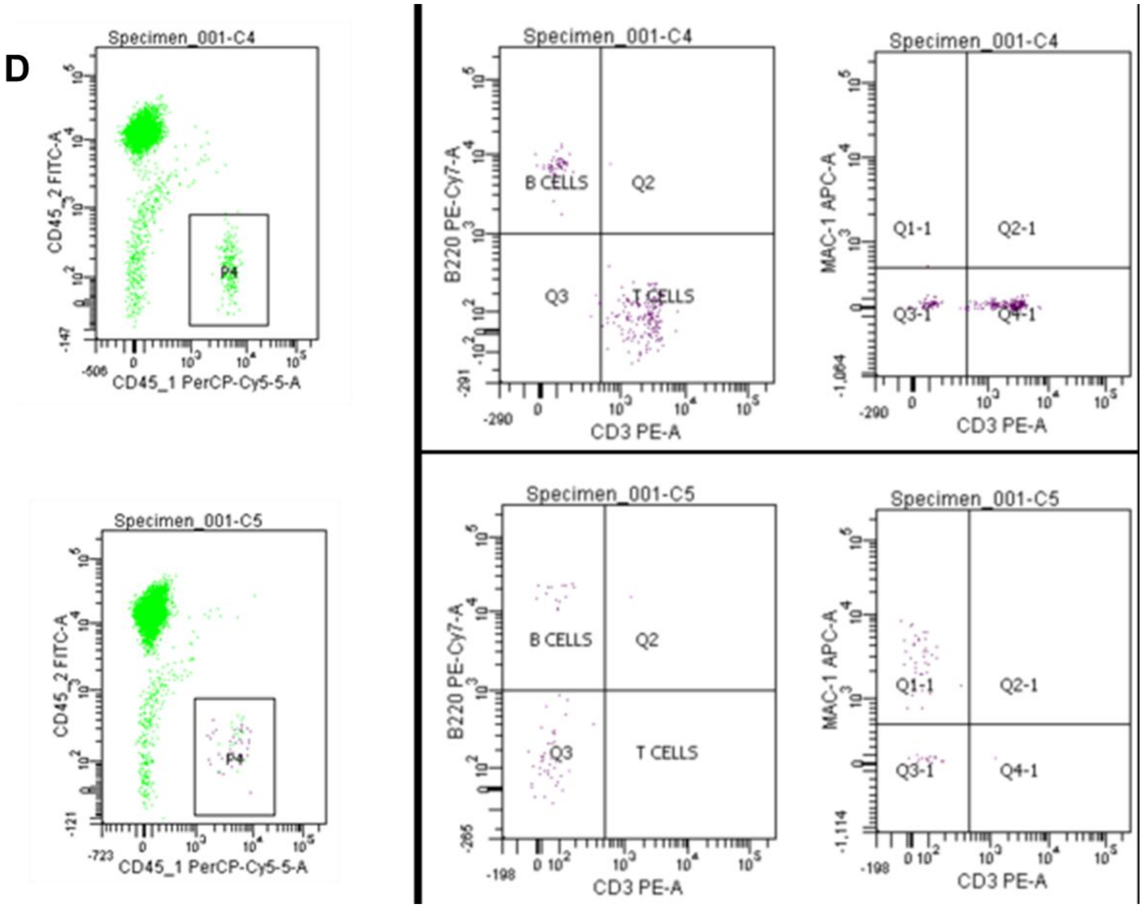

Figure S5. Representative flow plots of donor chimerism and lineage differentiation distribution in recipient bone marrow for compound **40** (**A**), XIE18-6 (**B**), DMSO-control (**C**) and no culture-control (**D**) groups.

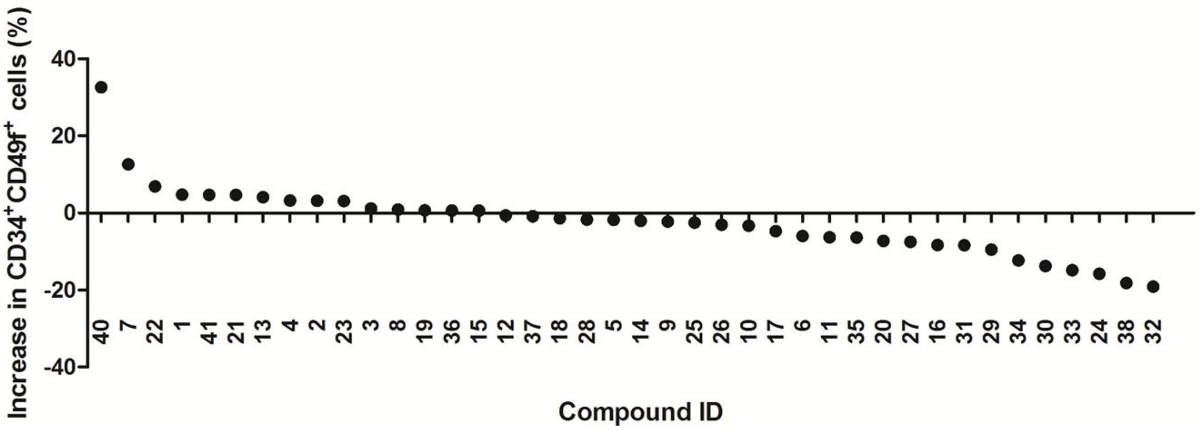

Figure S6. Primary screening hit identification of p18SMIs prompting expansion *ex vivo* of human hematopoietic stem cells.

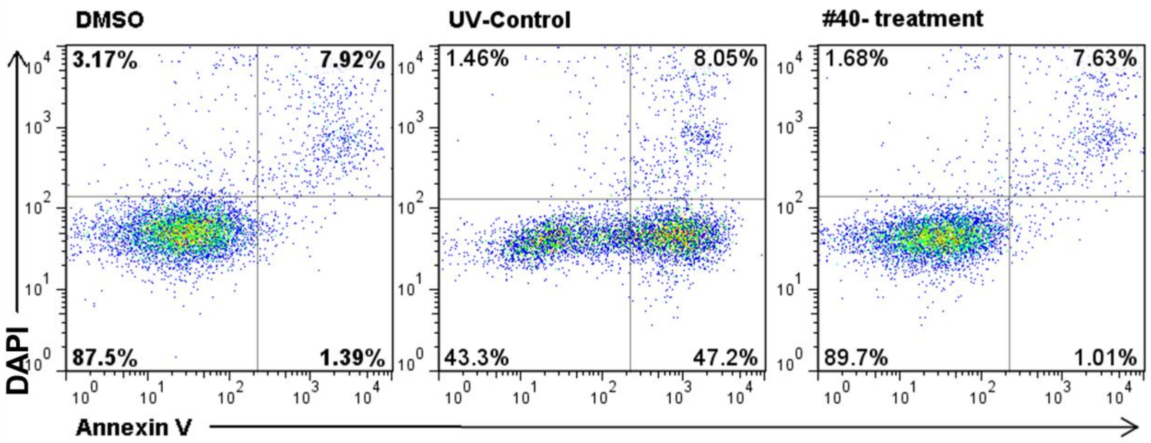

Figure S7. Representative figures of apoptosis of cultured c-Kit enriched BM cells which were cultured with cytokine plus 20 μM compound **40** or DMSO for 5 days. Uncultured bone marrow cells were irradiated by UV to serve as positive control. Apoptosis was checked by Annexin V staining on hematopoietic cells.

Table S1. Structure and activity to promote the expansion of HSCs for compounds XIE18-6, **1** and **2**

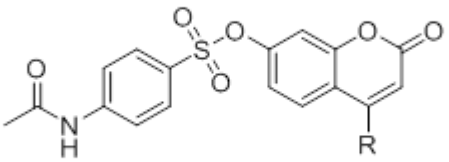

Compd. No.	Structure (-R)	ED_{50}, μM[a, b]	SD
XIE18-6	CH_3	0.10546	0.00361
**1**	H	0.09686	0.00216
**2**	*iso*-propyl	0.0610	0.00136

[a]The activity to promote the expansion of HSCs were evaluated using single hematopoietic stem cell *in vitro* culture assay. [b]The activity of lead compound XIE18-6 was evaluated in parallel with compounds **1** and **2** under the same conditions. Data are mean ± SD of all experiments of two or more performed in duplicate or triplicate.

Table S2. Structure and activity to promote the expansion of HSCs for compounds **3** – **14**

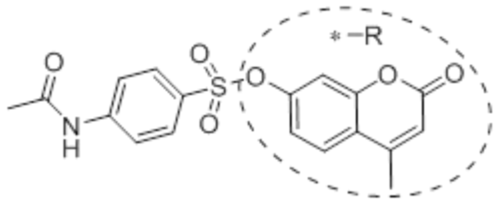

Compd. No.	Structure (-R)	ED_{50}, μM[a, b]	SD
**3**	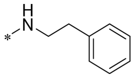	18.66	1.61
**4**	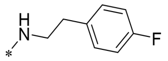	7.76	0.14
**5**	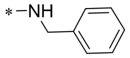	8.56	0.52
**6**	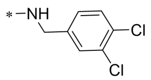	9.3	0.17
**7**	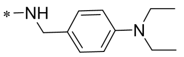	16.4	0.09
**8**	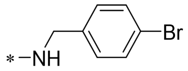	1.55	0.15
**9**	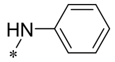	5.37	0.71
**10**	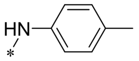	7.91	0.37
**11**	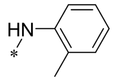	2.82	0.09
**12**	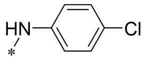	0.0259	0.00037
**13**	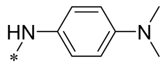	4.16	0.39
**14**	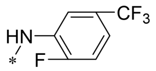	10.16	0.06
**XIE18-6**		0.10546	0.00361

[a]The activity to promote the expansion of HSCs were evaluated using single hematopoietic stem cell *in vitro* culture assay. [b]The activity of lead compound XIE18-6 was evaluated in parallel with compounds **3** – **14** under the same conditions. Data are mean ± SD of all experiments of two or more performed in duplicate or triplicate.

Table S3. Structure and activity to promote the expansion of HSCs for compounds 15 – 21

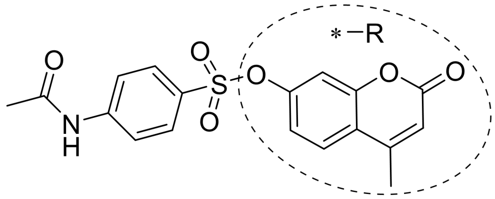

Compd. No.	Structure (-R)	ED_{50}, μM[a, b]	SD
**15**	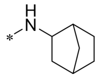	22.57	0.13
**16**	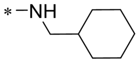	7.03	0.24
**17**	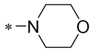	9.64	0.10
**18**	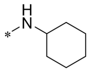	19.52	1.41
**19**	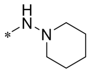	3.22	0.06
**20**	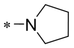	10.36	0.40
**21**	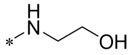	0.0107	0.00081
**XIE18-6**		0.10546	0.00361

[a]The activity to promote the expansion of HSCs were evaluated using single hematopoietic stem cell *in vitro* culture assay. [b]The activity of lead compound XIE18-6 was evaluated in parallel with compounds **15** – **21** under the same conditions. Data are mean ± SD of all experiments of two or more performed in duplicate or triplicate.

Table S4. Structure and activity to promote the expansion of HSCs for compounds **22** and **23**

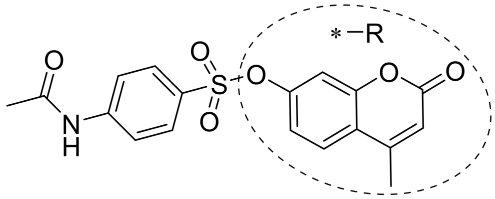

Compd. No.	Structure (-R)	ED_{50}, μM[a, b]	SD
**22**	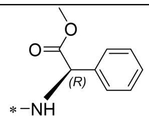	0.00729	0.00074
**23**	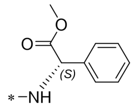	21.3	1.10
**XIE18-6**		0.10546	0.00361

[a]The activity to promote the expansion of HSCs were evaluated using single hematopoietic stem cell *in vitro* culture assay. [b]The activity of lead compound XIE18-6 was evaluated in parallel with compounds **22** and **23** under the same conditions. Data are mean ± SD of all experiments of two or more performed in duplicate or triplicate.

Table S5. Structure and activity to promote the expansion of HSCs for compounds **24** – **31**

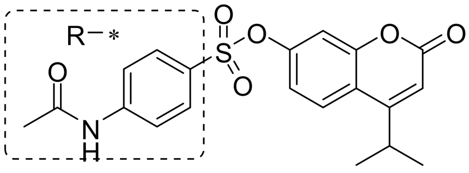

Compd. No.	Structure (-R)	ED_{50}, μM[a, b]	SD
**24**	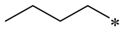	4.14	0.05
**25**	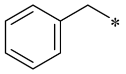	0.92	0.01
**26**	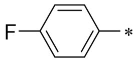	0.79	0.04
**27**	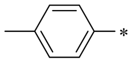	0.02087	0.00091
**28**	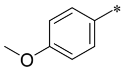	0.0595	0.00208
**29**	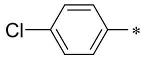	0.07822	0.00231
**30**	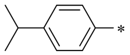	1.93	0.04
**31**	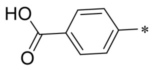	6.72	0.36
**XIE18-6**		0.10546	0.00361

[a]The activity to promote the expansion of HSCs were evaluated using single hematopoietic stem cell *in vitro* culture assay. [b]The activity of lead compound XIE18-6 was evaluated in parallel with compounds **15** – **21** under the same conditions. Data are mean ± SD of all experiments of two or more performed in duplicate or triplicate.

Table S6. Structure and activity to promote the expansion of HSCs for compounds **32** – **40**

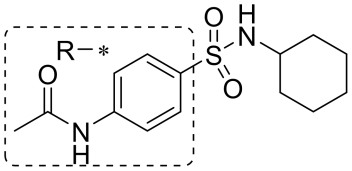

Compd. No.	Structure (-R)	ED_{50}, μM[a, b]	SD
**32**	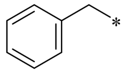	9.54	0.47
**33**	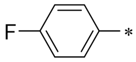	0.01967	0.00033
**34**	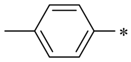	10.33	0.90
**35**	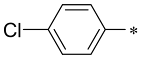	9.46	0.38
**36**	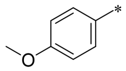	0.06283	0.00015
**37**	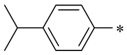	0.01005	0.00134
**38**	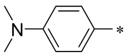	11.7	0.36
**39**	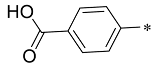	0.120	0.00020
**40**	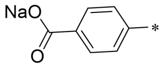	0.00521	0.00064
**XIE18-6**		0.10546	0.00361

[a]The activity to promote the expansion of HSCs were evaluated using single hematopoietic stem cell *in vitro* culture assay. [b]The activity of lead compound XIE18-6 was evaluated in parallel with compounds **15** – **21** under the same conditions. Data are mean ± SD of all experiments of two or more performed in duplicate or triplicate.

Table S7. Structure and activity to promote the expansion of HSCs for compound **41**

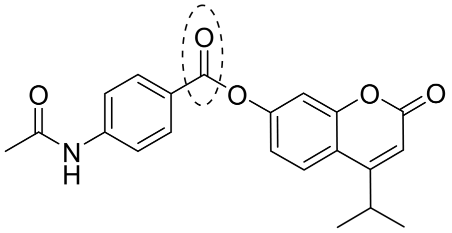

Compd. No.	ED_{50}, μM[a, b]	SD
**41**	0.00992	0.00071
**XIE18-6**	0.10546	0.00361

[a]The activity to promote the expansion of HSCs were evaluated using single hematopoietic stem cell *in vitro* culture assay. [b]The activity of lead compound XIE18-6 was evaluated in parallel with compound **40** under the same conditions. Data are mean ± SD of all experiments of two or more performed in duplicate or triplicate.
